# Proteomic alterations in early stage cervical cancer

**DOI:** 10.18632/oncotarget.24773

**Published:** 2018-04-06

**Authors:** Coşkun Güzel, Natalia I. Govorukhina, G. Bea A. Wisman, Christoph Stingl, Lennard J.M. Dekker, Harry G. Klip, Harry Hollema, Victor Guryev, Peter L. Horvatovich, Ate G.J. van der Zee, Rainer Bischoff, Theo M. Luider

**Affiliations:** ^1^ Laboratory of Neuro-Oncology, Clinical and Cancer Proteomics, Department of Neurology, Erasmus University Medical Center Rotterdam, Rotterdam 3015 CN, The Netherlands; ^2^ Department of Analytical Biochemistry, Center for Pharmacy, University of Groningen, Groningen 9713 AV, The Netherlands; ^3^ Department of Gynecologic Oncology, Cancer Research Center Groningen, University of Groningen, University Medical Center Groningen, Groningen 9713 GZ, The Netherlands; ^4^ Department of Pathology, University Medical Center Groningen, University of Groningen, Groningen 9713 GZ, The Netherlands

**Keywords:** LCM, cervical cancer, biomarker, PRM, proteomics

## Abstract

Laser capture microdissection (LCM) allows the capture of cell types or well-defined structures in tissue. We compared in a semi-quantitative way the proteomes from an equivalent of 8,000 tumor cells from patients with squamous cell cervical cancer (SCC, *n* = 22) with healthy epithelial and stromal cells obtained from normal cervical tissue (*n* = 13). Proteins were enzymatically digested into peptides which were measured by high-resolution mass spectrometry and analyzed by “all-or-nothing” analysis, Bonferroni, and Benjamini-Hochberg correction for multiple testing. By comparing LCM cell type preparations, 31 proteins were exclusively found in early stage cervical cancer (*n* = 11) when compared with healthy epithelium and stroma, based on criteria that address specificity in a restrictive “all-or-nothing” way. By Bonferroni correction for multiple testing, 30 proteins were significantly up-regulated between early stage cervical cancer and healthy control, including six members of the MCM protein family. MCM proteins are involved in DNA repair and expected to be participating in the early stage of cancer. After a less stringent Benjamini-Hochberg correction for multiple testing, we found that the abundances of 319 proteins were significantly different between early stage cervical cancer and healthy controls. Four proteins were confirmed in digests of whole tissue lysates by Parallel Reaction Monitoring (PRM). Ingenuity Pathway Analysis using correction for multiple testing by permutation resulted in two networks that were differentially regulated in early stage cervical cancer compared with healthy tissue. From these networks, we learned that specific tumor mechanisms become effective during the early stage of cervical cancer.

## INTRODUCTION

Cervical cancer is one of the most common cancers in women worldwide [1–3]. It is more prevalent in developing countries, where 83% of cases occur and where squamous cell cervical cancer (SCC) accounts for 15% of newly diagnosed cancers in women. In developed countries, it accounts for 3.6% of all new cancer cases [4]. It was shown that 99% of cervical cancers are linked to infection with “high-risk” strains of human papillomavirus (HPV) [5, 6]. HPV type 16 and 18 together are responsible for 70% of cervical cancers [7]. HPV infections are associated with the development of high-grade cervical intraepithelial neoplasia (CIN2/3), which may eventually lead to SCC [8]. It seems that the HPV E7 gene, which disrupts Rb function is devoid of genetic variants in precancer and cancer cases and strict conservation of 98 amino acids is critical for HPV16 carcinogenesis [9]. To gain a deeper insight into the relation between genomic alterations and the development of cervical cancer, Ojesina *et al.* [10, 11] performed exome and whole genome sequencing and showed relationships between recurrent somatic mutations, copy-number alterations, changes in transcript levels and gene alterations as a consequence of HPV integration, and the development of cervical cancer. Whole-genome sequencing showed that HPV integration disrupts HPV genes as well as probably nearby host genes. These disruptions can lead to specific mechanisms in cervical cancer. Linking genomics to proteomics data has recently led to new insights into these mechanisms in cancer development [12, 13]. Laser capture microdissection (LCM) is a technique that allows the capture of different cell types or well-defined structures in tissue for subsequent genomics and proteomics analyses [14, 15]. Notably tissue from cancer patients has been analyzed by proteomics following this LCM technique [16–21]. Although this approach is very challenging due to the minimal amount of tissue material available for analysis, it was shown previously that a few hundred proteins can be identified from about 12,000 isolated cells from cervical cytological specimen [22]. We compared by semi-quantitative analyses the protein abundances of 4,488 proteins based on one peptide using approximately 8,000 cells obtained from LCM-derived cervical cancer tissue with healthy cervical epithelium or stroma from women with a normal cervix. Parallel Reaction Monitoring (PRM) was applied to confirm selected differential proteins found by the shotgun proteomics approach. Data was subjected to Ingenuity Pathway Analysis (IPA) linking published genomics data to proteomics data generated in this study. From the networks acquired, we learned that specific tumor mechanisms become effective during the early stage of cervical cancer.

## RESULTS

The study included patients with squamous cell cervical cancer (SCC, *n* = 22) and controls collected from patients who have undergone a hysterectomy for non-malignant reasons (*n* = 13) (see Table 1).

**Table 1:** Overview of all squamous cervical cancer patients and healthy controls from which tissue was obtained

Sample number	Age	FIGO stage
**Early stage cervical cancer I/II**		
1218	50	Ib2
1230	44	IIb
1239	43	Ib1
1291	34	Ib2
1298	80	Ib2
2110	77	IIb
2146	46	Ib2
2163	39	Ib1
2180	74	IIb
2246	73	Ib1
2252	44	Ib2
**Late stage cervical cancer III/IV**		
1225	58	IIIb
1348	71	IIIa
1685	26	IIIb
1966	75	IVb
1981	68	IIIb
1998	72	IIIb
2008	84	IIIa
2087	49	IIIb
2247	76	IIIb
2265	83	IVb
2297	52	IVa
**Healthy cervical epithelium**		**Type**
1247	43	hypermenorrhea/meno-/metrorrhagia
1262	51	prolapse
1315	36	prolapse
1477	41	uterine leiomyoma
1516	39	dysmenorrhea
1525	41	hypermenorrhea/meno-/metrorrhagia
1542	46	prolapse
1826	45	uterine leiomyoma
1849	51	hypermenorrhea/meno-/metrorrhagia
1936	41	hypermenorrhea/meno-/metrorrhagia
1948	60	prolapse
2000	41	uterine leiomyoma
2001	49	uterine leiomyoma

### Protein profiling by high-resolution mass spectrometry

**Figure 1:**
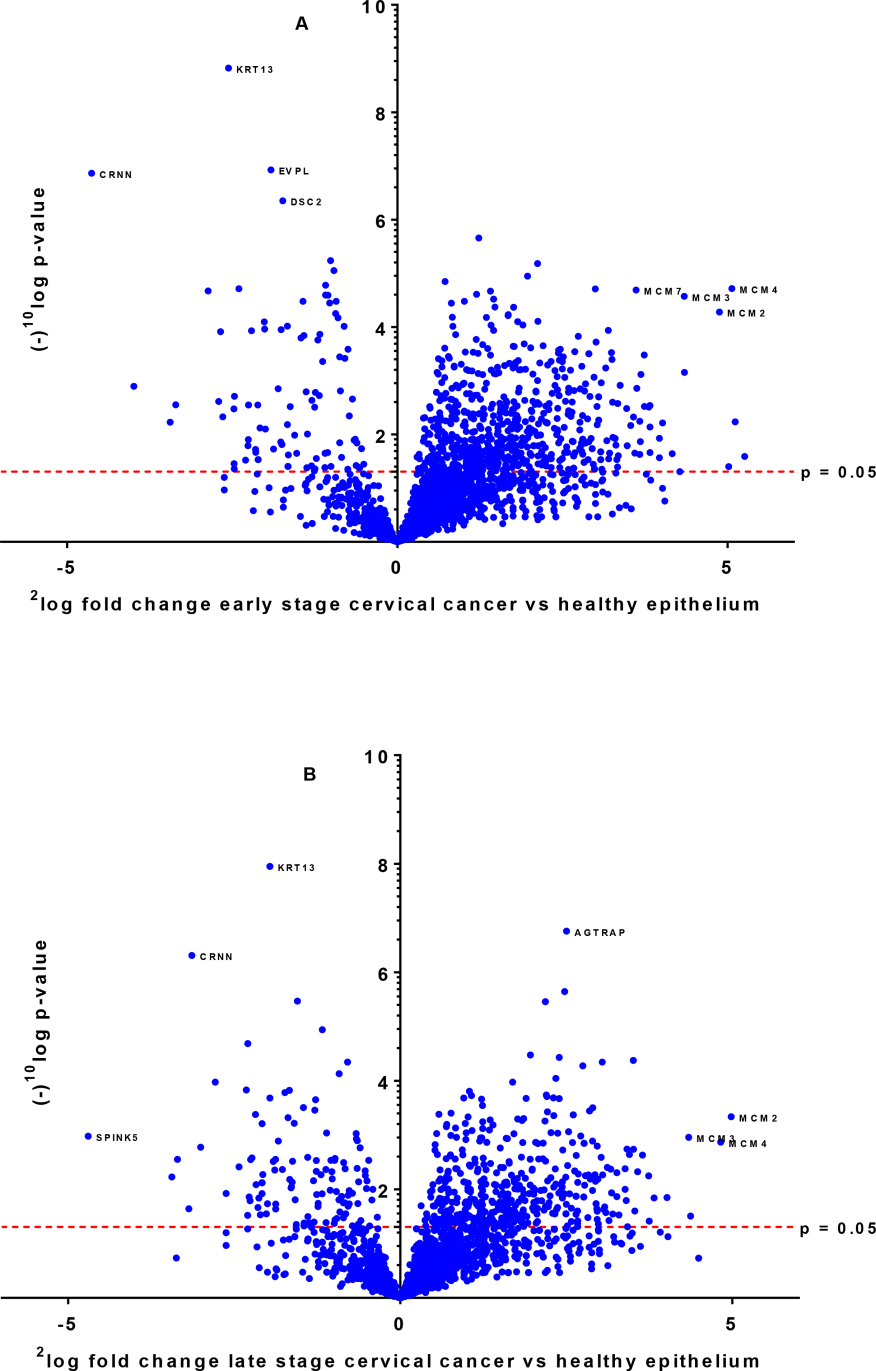
Differentially expressed proteins between early stage (**A**) and late stage (**B**) cervical cancer compared to healthy epithelium illustrated by a volcano plot. The x-axis represents the ^2^log fold-change and y-axis the (−)^10^log *p*-value. Examples of proteins that are differentially expressed with high significance are indicated with their names.

By shotgun proteomics, the technical reproducibility of a tissue lysate digest in triplicate showed an overlap of identified proteins of 65%. The methodological overlap for three independently prepared identical LCM samples (performing digestion and microdissection) was 76%. Analysis of eleven early stage and eleven late stage SCC, thirteen healthy epithelium and healthy stroma samples by nano-LC-MS/MS resulted in a total of 2,989 identified proteins with a minimum number of two peptides, an FDR rate of 0.4% and an FDR rate of 0.04% for peptides based on 262,136 MS/MS spectra. On average 1,700 proteins per sample (1,296–2,189) were identified. Interestingly, levels of members of the Minichromosome Maintenance (MCM) family (*MCM2*, *MCM3*, *MCM4*, *MCM5*, *MCM6*, *MCM7*) [23, 24] were found to be highly significantly increased in cervical cancer tissue from early and late stage patients as compared to healthy tissue. A volcano plot illustrates the difference in abundance of proteins in the comparison of both early stage and late stage cervical cancer with the healthy epithelium (Figure 1A and 1B).

Data were analyzed by three analysis approaches to find proteins which were highly discriminative (“all-or-nothing” method, Bonferroni and a third less restrictive method Benjamini-Hochberg). In the most stringent “all-or-nothing” analysis, we searched for proteins being present in the early stage cervical cancer group (it was allowed that these proteins are presented in late stage cervical cancer as well) and not in healthy epithelium or stroma. This approach showed that 31 proteins (*p* < 0.05) were discriminative between early stage cervical cancer and healthy epithelium (Table 2A). With this analysis, only *MCM4* of the MCM2-7 family was observed in early and late stage cervical cancer and not detectable in healthy epithelium and stroma cells. On the other hand, three proteins, *ENDOU*, *MT-ND4* and *RDH12* were exclusively present in healthy epithelium (in at least six out of thirteen samples). For stroma, six proteins *TNXB*, *COL21A1*, *OLFML1*, *FMOD*, *HSPB6* and *ABI3BP* were found (in at least six out of thirteen samples). After correction for multiple testing using the Bonferroni analysis (*p* = 9.88 ´ 10^−6^), 30 proteins (Table 2B) were significantly different in the comparison of early stage cervical cancer tissue and healthy epithelium, while thirteen proteins (Supplementary Table 1) were significant different between late stage cervical cancer tissue and healthy epithelium. Using the Benjamini-Hochberg correction (FDR = 5%) for multiple testing as the less stringent method, the abundances of 319 proteins (Supplementary Table 2) were found to be significantly different between early stage cervical cancer and healthy epithelium. Comparison of late stage cervical cancer with healthy epithelium resulted in 140 proteins (Supplementary Table 3) having significantly different levels.

**Table 2:** List of significant different proteins between early stage cervical cancer and healthy epithelium based on “all-or-nothing principle” (A; *n* = 31) and after Bonferroni analysis (B; *n* = 30)

A. “All-or-nothing” analysis			
Protein name	Gene name	*p*-value	^2^log fold–change
DNA replication licensing factor MCM4	MCM4	1.92E-05	5.1
Protein S100-P	S100P	3.13E-04	2.9
DNA polymerase delta catalytic subunit	POLD1	3.13E-04	2.9
DnaJ homolog subfamily C member 13	DNAJC13	1.21E-03	3.4
Replication factor C subunit 2	RFC2	1.21E-03	3.4
Inactive tyrosine-protein kinase 7	PTK7	1.42E-03	2.8
Synembryn-A	RIC8A	1.42E-03	2.8
Cytospin-B	SPECC1	1.88E-03	2.4
U6 snRNA-associated Sm-like protein LSm2	LSM2	1.88E-03	2.4
ADP-dependent glucokinase	ADPGK	1.88E-03	2.4
Eyes absent homolog 3	EYA3	1.96E-03	2.9
Polypeptide N-acetylgalactosaminyltransferase 2	GALNT2	1.99E-03	3.0
PEST proteolytic signal-containing nuclear protein	PCNP	1.99E-03	3.0
HLA class II histocompatibility antigen, DRB1-16 beta chain	HLA-DRB1	2.00E-03	4.2
E3 ubiquitin-protein ligase DTX3L	DTX3L	3.00E-03	3.7
Importin subunit alpha-2	KPNA2	3.08E-03	3.8
CTP synthase 1	CTPS1	4.58E-03	2.6
Prolyl 3-hydroxylase 1	LEPRE1	4.58E-03	2.6
Peptidyl-tRNA hydrolase 2, mitochondrial	PTRH2	4.58E-03	2.6
15 kDa selenoprotein	SEP15	4.58E-03	2.6
Intercellular adhesion molecule 1	ICAM1	4.75E-03	3.6
Acyl-coenzyme A thioesterase 9, mitochondrial	ACOT9	5.42E-03	3.0
Poly [ADP-ribose] polymerase 9	PARP9	5.66E-03	3.7
Nuclear pore membrane glycoprotein 210	NUP210	6.09E-03	4.0
Carcinoembryonic antigen-related cell adhesion molecule 5	CEACAM5	7.27E-03	3.8
Structural maintenance of chromosomes flexible hinge domain-containing protein 1	SMCHD1	1.19E-02	2.9
DBIRD complex subunit ZNF326	ZNF326	1.19E-02	2.9
E3 ubiquitin-protein ligase BRE1A	RNF20	1.43E-02	2.8
Mitochondrial Rho GTPase 2	RHOT2	1.43E-02	2.8
Sterile alpha motif domain-containing protein 9	SAMD9	2.17E-02	3.8
Myeloperoxidase	MPO	3.96E-02	5.0
**B. Bonferroni correction for multiple testing**			
**Protein name**	**Gene name**	***p*-value**	**^2^log fold–change**
Keratin, type I cytoskeletal 13^*^	KRT13	1.50E-09	−2.6
Envoplakin	EVPL	1.18E-07	−1.9
Cornulin^*^	CRNN	1.37E-07	−4.6
Desmocollin-2^*^	DSC2	4.47E-07	−1.7
Acidic leucine-rich nuclear phosphoprotein 32 family member A	ANP32A	1.57E-06	1.4
DNA topoisomerase 1	TOP1	6.56E-06	2.1
Protein disulfide-isomerase TMX3	TMX3	1.13E-05	2.0
Protein disulfide-isomerase A3	PDIA3	1.42E-05	0.7
DNA replication licensing factor MCM4	MCM4	1.92E-05	5.1
DNA replication licensing factor MCM6^*^	MCM6	1.95E-05	3.0
DNA replication licensing factor MCM7^*^	MCM7	2.04E-05	3.6
Desmoglein-1	DSG1	2.12E-05	−2.9
Ribosome-binding protein 1	RRBP1	2.15E-05	1.4
Exportin-2	CSE1L	2.45E-05	1.2
DNA replication licensing factor MCM3	MCM3	2.68E-05	4.3
Heterogeneous nuclear ribonucleoproteins A2/B1	HNRNPA2B1	3.31E-05	1.0
Keratin, type II cytoskeletal 5	KRT5	3.55E-05	−1.0
78 kDa glucose-regulated protein	HSPA5	3.60E-05	0.8
Calreticulin	CALR	4.23E-05	1.5
Nucleoprotein TPR	TPR	4.28E-05	1.8
DNA replication licensing factor MCM2	MCM2	5.24E-05	4.9
Endoplasmic reticulum resident protein 29^*^	ERP29	7.93E-05	1.8
Nuclear pore complex protein Nup155	NUP155	9.16E-05	1.9
Phospholipase A-2-activating protein	PLAA	1.15E-04	3.2
Flap endonuclease 1^*^	FEN1	1.49E-04	2.7
Tryptophan--tRNA ligase, cytoplasmic	WARS	1.90E-04	3.0
Pre-mRNA-processing factor 6	PRPF6	2.99E-04	3.2
Protein S100-P	S100P	3.13E-04	2.9
DNA polymerase delta catalytic subunit	POLD1	3.13E-04	2.9
Serpin H1	SERPINH1	3.30E-04	3.7

Zero counts were converted to 0.125 to enable log calculations. Supplementary Table 1 shows a complete list of proteins assigned to late stage cervical cancer analyzed by Bonferroni analysis (here indicated with an asterisk (^*^)) and individual comparison of early and late stage cervical cancer with healthy epithelium using Benjamini-Hochberg as shown in Supplementary Tables 2 and 3, respectively.

**Figure 2:**
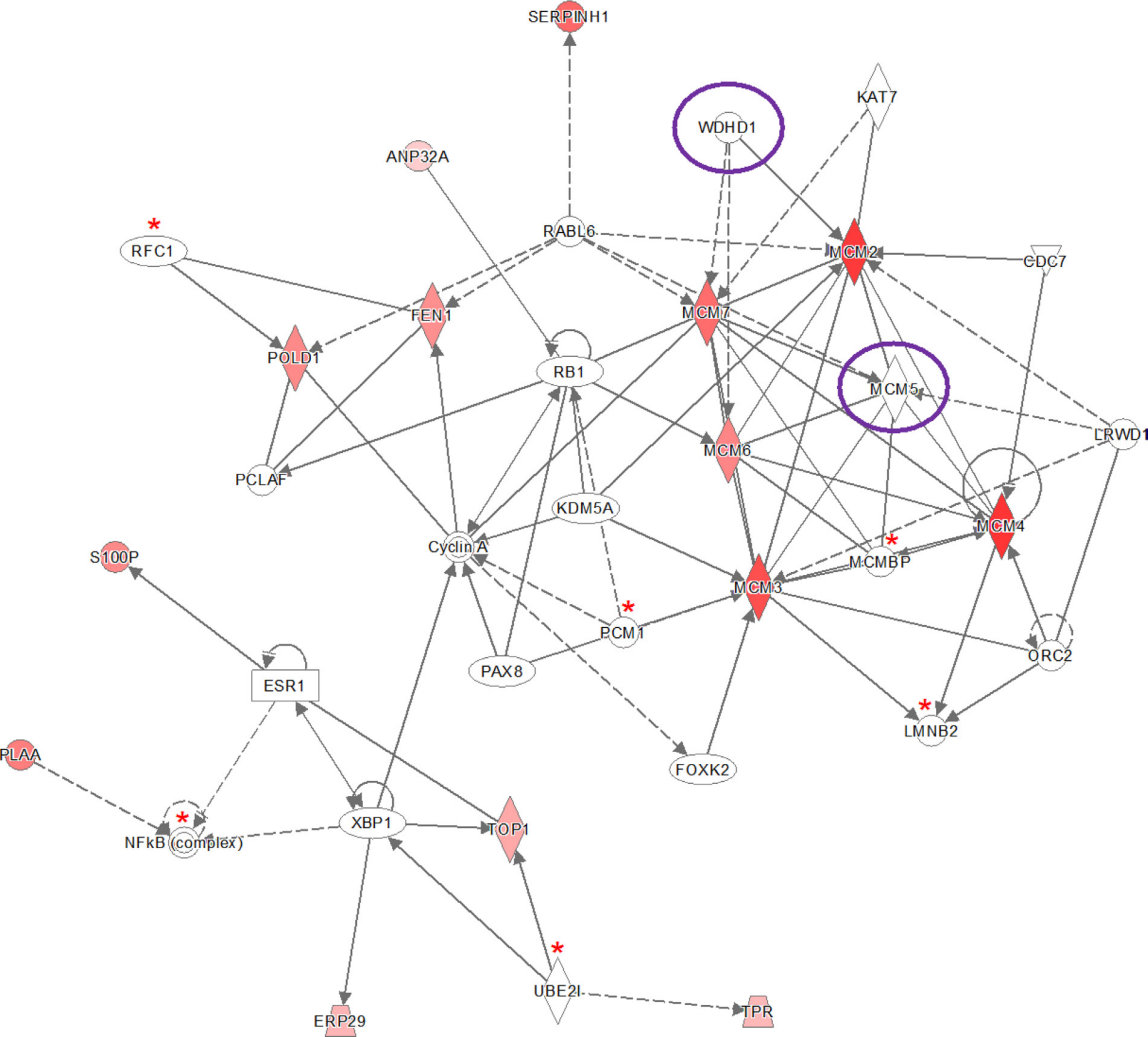
Ingenuity pathway analysis (IPA) of the 30 significantly up- and down-regulated proteins (after Bonferroni analysis) for early stage cervical cancer versus healthy controls Pathway analysis indicated that the network “DNA Replication, Recombination, and Repair”, containing fourteen out of the 30 significant proteins, is up-regulated. The network itself consists of 35 proteins. The up-regulated proteins in early stage cervical cancer are marked red, while those that that were down-regulated were not identified in this pathway. The intensity of the color relates to fold-change. Proteins indicated with a red asterisk were found by LCM, although not differential. Benjamini-Hochberg analysis of the same data resulted in two more proteins (encircled in purple) belonging to this network (*MCM5* and *WDHD1*). The symbols shown in the network are explained at http://www.qiagenbioinformatics.com/products/ingenuity-pathway-analysis

The IPA software tool was used to find networks in which the identified differentially abundant proteins might be involved. Fourteen out of the 30 proteins that were significant for early stage cervical cancer after Bonferroni analysis were classified to the cell cycle control network (“DNA Replication, Recombination, and Repair”), which comprises a total of 35 proteins (Figure 2) and had a network score of 30. This score was above the upper 95% confidence level of the mean threshold (i.e. upper 95% Cl mean of 27.2) that was calculated by permutation from randomly taken 30 proteins of UniProt database. Six proteins from the network (*LMNB2*, *MCMBP*, *NF-kB complex*, *PCM1*, *RFC1*, *UBE2l*) were identified but not differential by the LCM approach with at least one peptide. Two proteins belonging to this network (*MCM5* and *WDHD1*) were found by Benjamini-Hochberg analysis of the same data.

**Figure 3:**
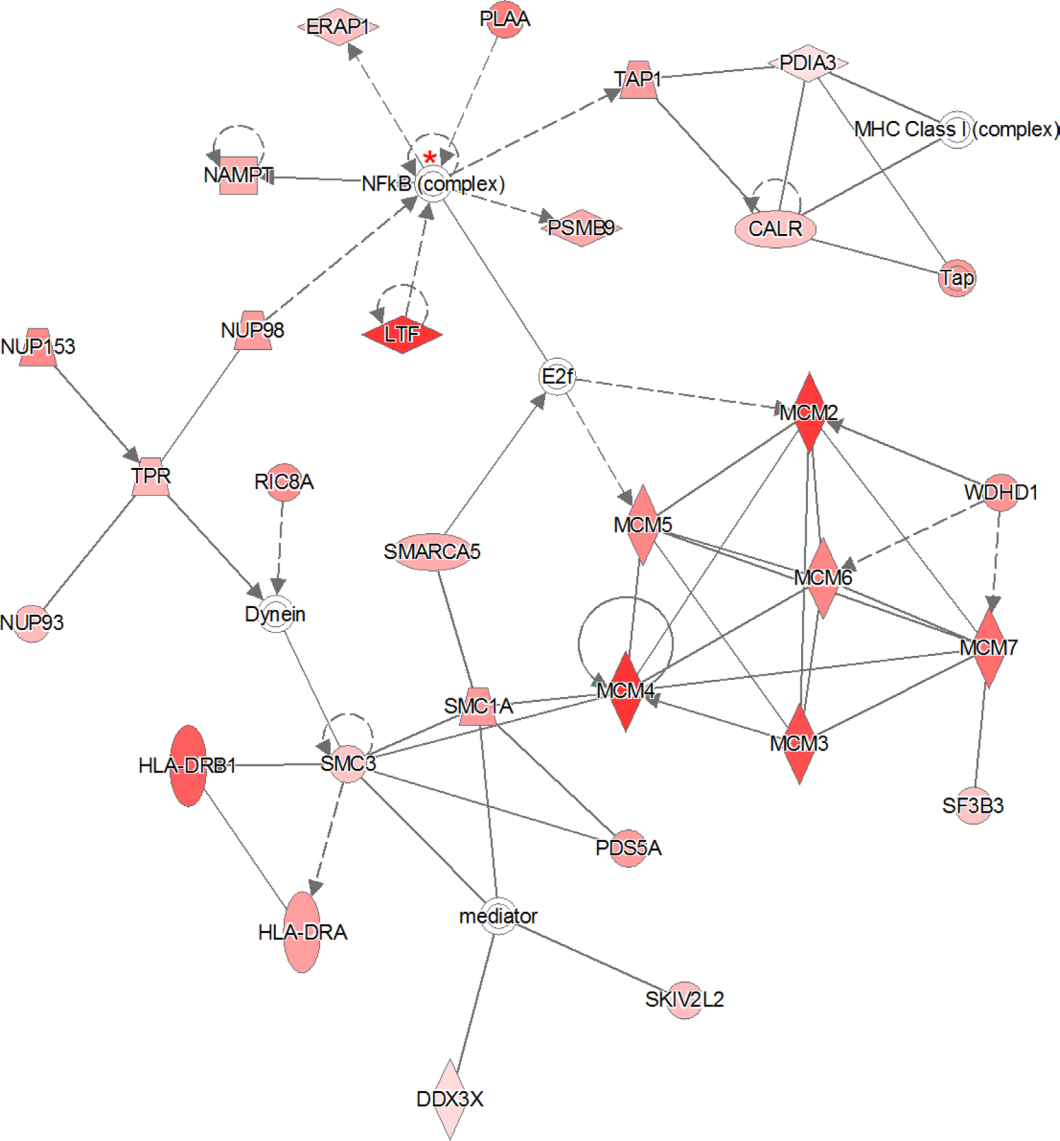
The 319 significantly proteins (up- and down-regulated) that were found by Benjamini-Hochberg analysis for early stage cervical cancer were applied to the IPA analysis tool The pathway analysis resulted in two networks with identical scores that passed the threshold. The indicated network matched to “DNA Replication, Recombination, and Repair” containing 29 out of the 319 significant proteins. The network itself consists of 35 proteins. The up-regulated proteins in early stage cervical cancer are marked red, while those that that were down-regulated were not identified in this pathway. The intensity of the color relates to fold-change. The NF-κB complex protein indicated with a red asterisk was identified once (99% protein- and 95% peptide probability with at least one peptide) by LCM, although not differential. The second network that passed the permutation background score is represented in Figure 4. The symbols shown in the network are explained at http://www.qiagenbioinformatics.com/products/ingenuity-pathway-analysis

**Figure 4:**
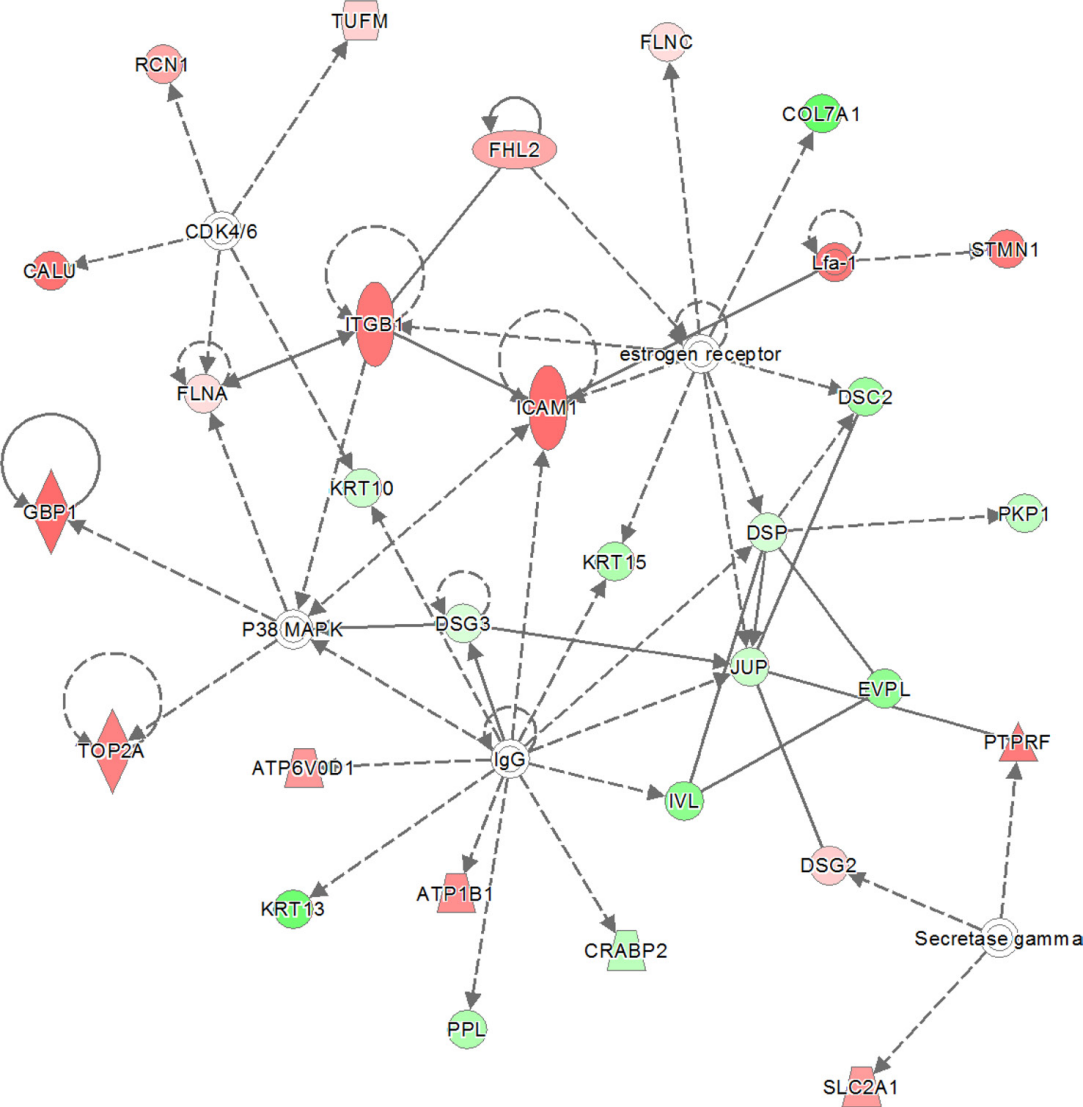
The second network with identical score as shown in previous network (Figure 3) The 319 significantly proteins (up- and down-regulated) that were found by Benjamini-Hochberg analysis for early stage cervical cancer were applied to the IPA analysis tool. The pathway analysis resulted in the finding of the network “Cardiac Arrythmia, Cardiovascular Disease, Organismal Injury and Abnormalities” containing 29 out of the 319 significant proteins. The network itself consists of 35 proteins. The up-regulated proteins in early stage cervical cancer are marked red and those up-regulated in healthy epithelial cells are marked green. The intensity of the color (red or green) relates to fold-change. Interestingly, almost 50% (13 out of 29) of proteins identified were down-regulated. The symbols shown in the network are explained at http://www.qiagenbioinformatics.com/products/ingenuity-pathway-analysis

**Figure 5:**
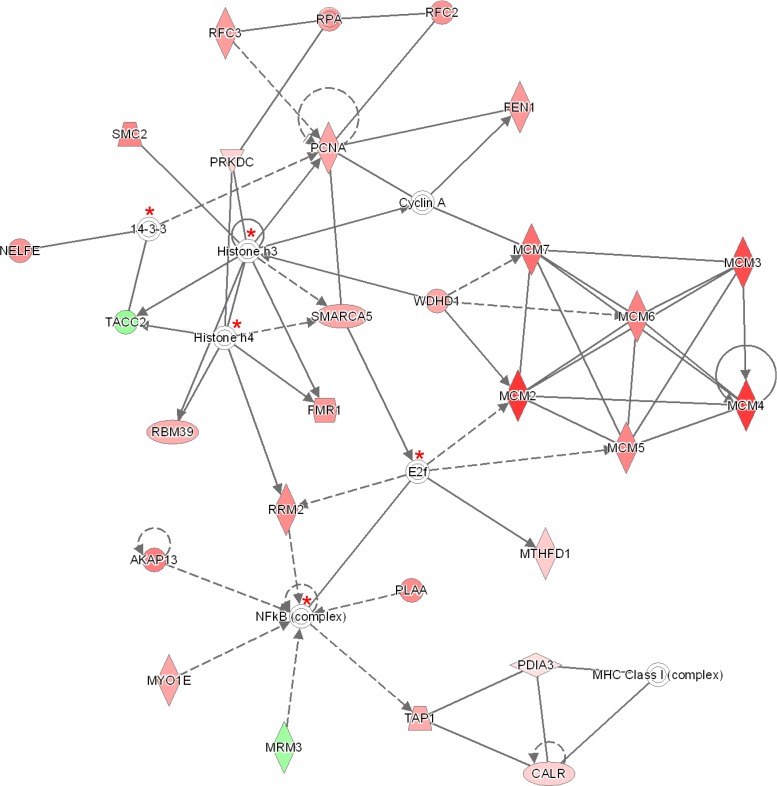
The 140 significantly proteins (up- and down-regulated) that were found by Benjamini-Hochberg analysis for late stage cervical cancer were applied to the IPA analysis tool The pathway analysis resulted in the finding of the network “DNA Replication, Recombination, and Repair” containing 27 out of the 140 significant proteins. The network itself consists of 35 proteins. The up-regulated proteins in late stage cervical cancer are marked red and two proteins which were up-regulated in healthy epithelial cells are indicated with a green color. The intensity of the color (red or green) relates to fold-change. Proteins (*n* = 5) indicated with a red asterisk have been identified minimal once (99% protein- and 95% peptide probability with at least one peptide) by LCM, although not differential. The symbols shown in the network are explained at http://www.qiagenbioinformatics.com/products/ingenuity-pathway-analysis

The “all-or-nothing” analysis and Bonferroni analysis between late stage cervical cancer and healthy epithelium had scores below the upper 95% confidence level of the mean threshold (i.e. 22 < 28.9 and 8 < 11.8, respectively) and for this reason the created networks were not further investigated. On the other hand, for the Benjamini-Hochberg analysis two networks (i.e. “DNA Replication, Recombination, and Repair” and “Cardiac Arrhythmia, Cardiovascular Disease, Organismal Injury and Abnormalities”; see Figure 3 and Figure 4, respectively) were found with an identical score that passed the threshold (i.e. 43 > 41.4). For the other group, comparing late stage cervical cancer with healthy epithelium one network (also related to “DNA Replication, Recombination, and Repair”; see Figure 5) identified by the Benjamini-Hochberg analysis exceeded the threshold score of 40.8 (i.e. 48 > 40.8). Proteins indicated in the “DNA Replication, Recombination, and Repair” network identified by IPA (i.e. Figure 2, Bonferroni analysis; Figure 3 and 5, Benjamini-Hochberg analysis) showed an overlap close to 30% of all network proteins mentioned. The other network (“Cardiac Arrhythmia, Cardiovascular Disease, Organismal Injury and Abnormalities”, Figure 4) consisted of almost 50% of down-regulated proteins that did not show any overlap with the first network mentioned (Figure 3). An overview of the different analyses achieved by comparison of individual early and late stage cervical cancer with healthy epithelium is displayed in Table 3.

**Table 3:** Overview of different types of analyses between early stage cervical cancer (EC) with healthy epithelium (H) and late stage cervical cancer (LC) with healthy epithelium

Type of analysis	EC versus H		Threshold score by permutation (*n* = 10) related to EC versus H	Number of networks found above threshold score related to EC versus H	LC versus H		Threshold score by permutation (*n* = 10) related to LC versus H	Number of networks found above threshold score related to LC versus H
	Number of differential proteins	IPA score^***^			Number of differential proteins	IPA score^***^		
“all-or-nothing”	31	22	28.9 (18.1–28.9)^*^	0	-	-	- ^**^	- ^**^
Bonferroni	30	30	27.2 (17.8–27.2)^*^	1	13	8	11.8 (3.2–11.8)^*^	0
Benjamini-Hochberg	319	43	41.4 (37.1–41.1)^*^	2	140	48	40.8 (28.0–40.8)^*^	1

After applying the significant differential proteins analyzed by three analyses i.e. “all-or-nothing”, Bonferroni and Benjamini-Hochberg to IPA, the network scores were matched with a threshold score determined by permutation. This score was obtained after a permutation test (*n* = 10) according to the number of proteins identified by the three analyses and defined as the upper 95% CL of mean value.

Network scores above this value were selected for discussion.

^*^The lower and upper 95% CI of mean.

^**^Not applied for LC vs H analyzed by the “all-or-nothing” analysis.

^***^IPA scores were calculated by IPA using right-tailed Fisher's Exact test.

**Figure 6:**
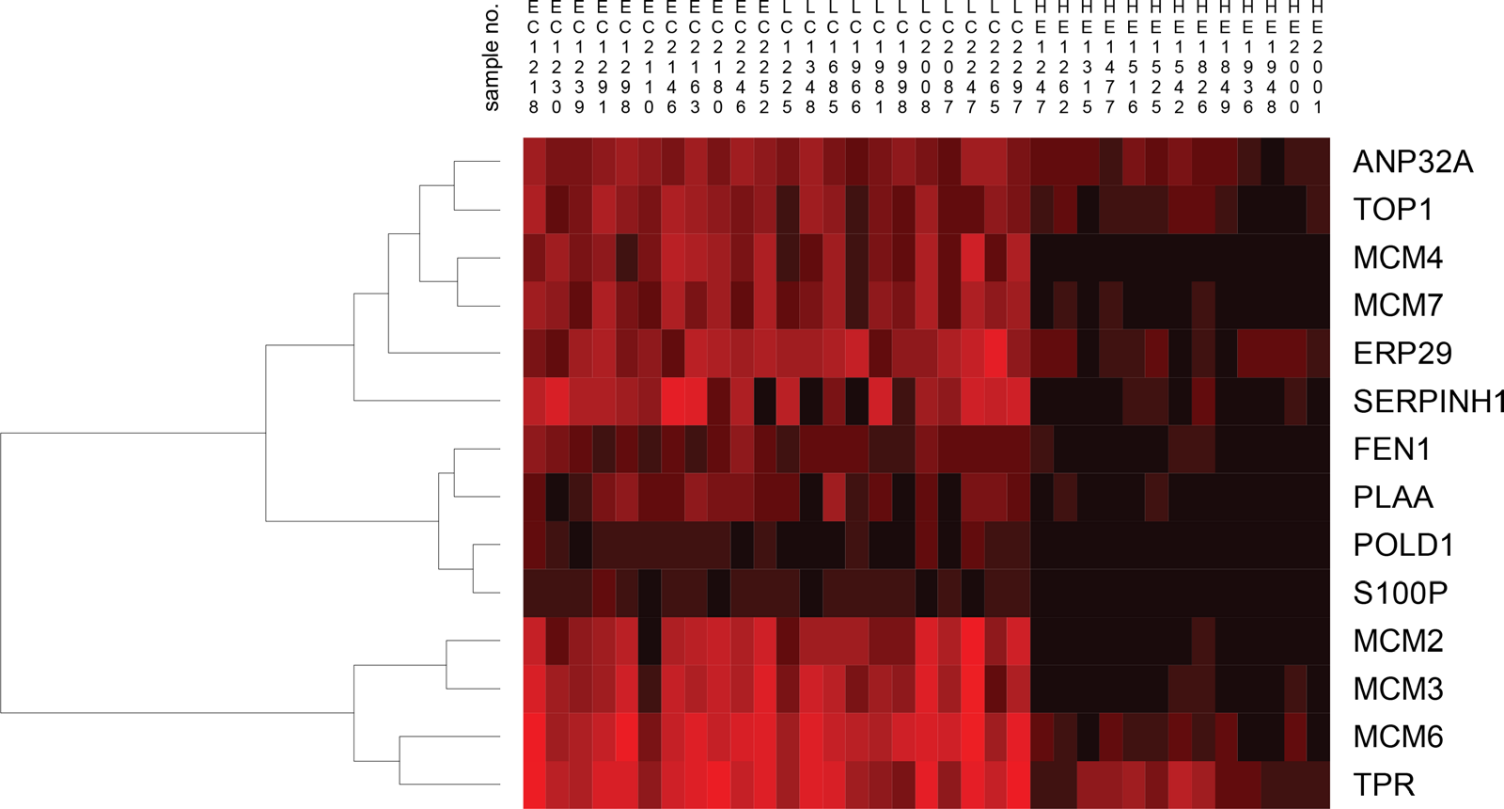
Hierarchical clustering of fourteen proteins found by the IPA tool related to early stage cervical cancer (EC), late stage cervical cancer (LC) and healthy epithelium (HE) visualizes the heterogeneity among individual samples. For the early and late stage cervical cancer group, clustering of MCM proteins was readily observed and showed high similarity between samples. Interestingly, the *MCM2*, *MCM3*, *MCM6* and *MCM4*, *MCM7* clustered in two different clusters. For the healthy epithelium group, the result was different compared to early and late stage cervical cancer group in which only *MCM6* from the MCM2-7 family clustered separately. The abundance levels of proteins were indicated with red (high) and black (low). Sample numbers correspond to those shown in Table 1.

Hierarchical clustering of the differentially abundant fourteen proteins found in the “DNA, Replication, Recombination and Repair Network” by IPA analysis of the Bonferroni analysis resulted in clusters of proteins showing similarities between the early and late stage cervical cancer, while for the healthy epithelium group this was significantly different (Figure 6).

### Protein profiles in early stage cervical cancer tissue by LCM with HeLa, U87 and HEK293 cell lines

From the 31 proteins (Table 2A) that were exclusively found by the “all-or-nothing” analysis in early stage cervical cancer and that all meet an extra criterion: ^2^log fold-change > 1, 30 were up-regulated in late stage cervical cancer tissue, 24 in the HeLa cell line, eleven in U87 and nine in HEK293 cells. From the 30 significantly different proteins selected after using Bonferroni analysis (Table 2B), according to identical criterion as mentioned above, 22 were up-regulated in both early stage cervical cancer and in the HeLa cell line and nineteen in late stage cervical cancer tissue. Fifteen out of the 30 significantly different proteins were up-regulated in the U87 cell line, while fourteen proteins were up-regulated in HEK293 cells. From the 319 significantly different proteins selected after using Benjamini-Hochberg correction (Supplementary Table 2) using identical criterion, 249 were up-regulated in early stage cervical cancer, 210 in the late stage cervical cancer, 244 in the HeLa cell line, 163 in the U87 cell line and 109 were up-regulated in the HEK293 cell line.

**Figure 7:**
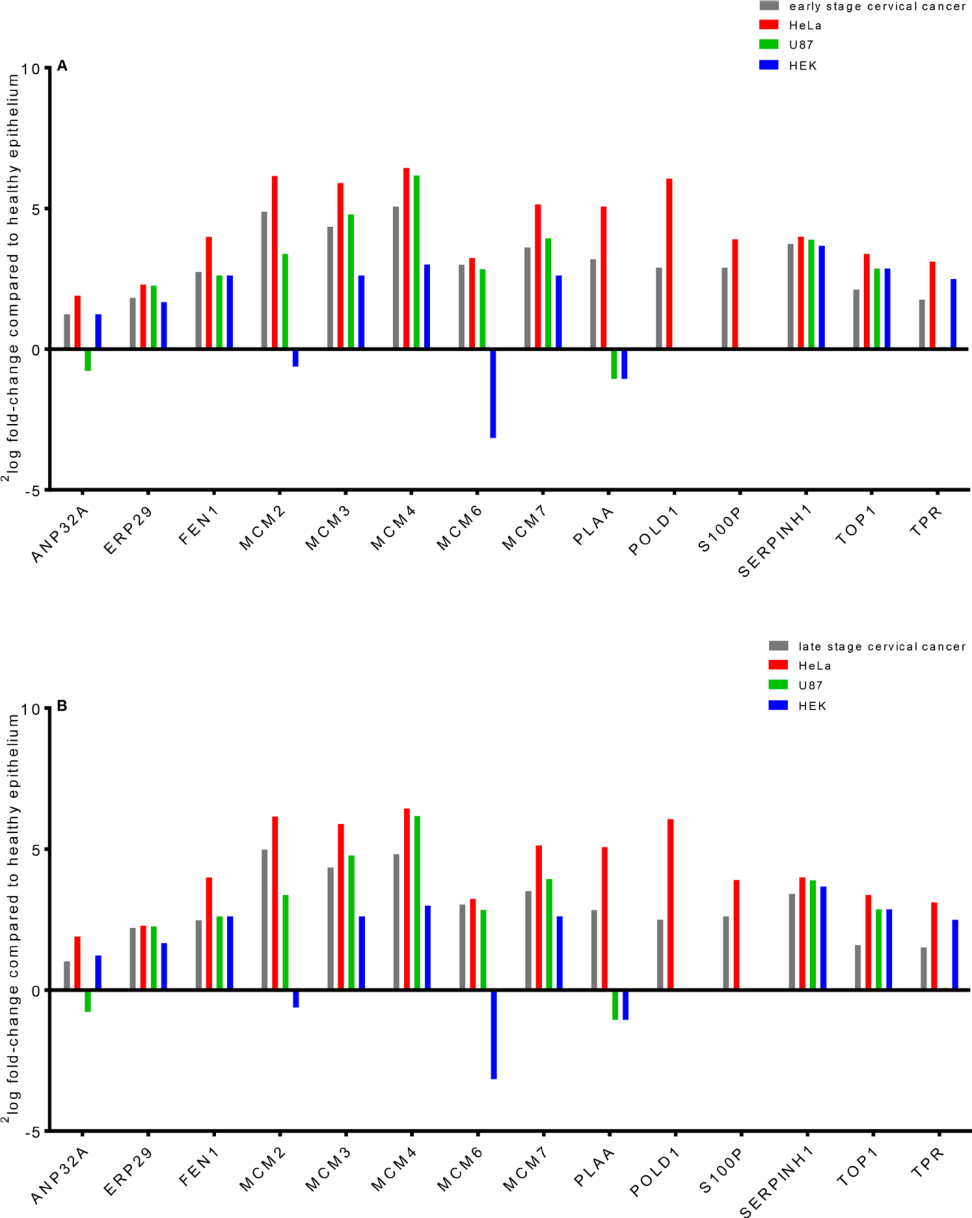
Laser microdissected healthy epithelial cells were compared in a semi-quantitative way with early (**A**) and late (**B**) stage cervical cancer, a cervical cancer derived cell line (HeLa) and two cell lines that were derived from brain tumor (U87) and normal embryonal kidney tissue (HEK). Most of the fourteen differential proteins which were from the network behave similar between healthy epithelium and various tumor types, except for a few. The proteins *PLAA*, *POLD1* and *S100P* were only observed in cervical cancer and HeLa digests when comparing to healthy epithelium (zero counts were converted into 0.125 to allow logarithm calculation). It was shown that MCM proteins in cervical cancer can have a 32-fold increase (^2^log fold-change of 5, e.g. *MCM4*) in abundance compared to healthy epithelium.

Three out of the 35 proteins (Figure 2) that were related to the network “DNA Replication, Recombination, and Repair” were significantly up-regulated (^2^log fold-change > 1) in both early and late stage cervical cancer and in the HeLa cell line compared to healthy epithelium (*NF-κB complex*, *PLAA*, *POLD1*, *S100P* and *WDHD1*), but were absent in the other two cell lines (U87 and HEK293). In Figure 7, the presence or absence of the differential proteins found by Bonferroni analysis only related to this network observed in early and late stage cervical cancer is illustrated.

Furthermore, comparison of results derived from all proteins identified with LCM-derived tumor cells from early and late stage cervical cancer and HeLa cell line (following criterion: ^2^log fold-change relative to healthy epithelium ≥2.5), with non-cervical U87 and HEK293 cell lines showed nineteen highly significantly up-regulated proteins. Following the same criterion, fourteen proteins were found to be exclusively discriminative for the LCM dissected tumor cells only (Table 4).

**Table 4:** Up- or down-regulated of all identified proteins calculated by 2log fold-changes (all compared to healthy epithelium) for LCM-derived cervical cancer cells and for HeLa cell line compared to other cell lines U87 and HEK293

**A.**	**Gene name**	**EC (LCM)**	**LC (LCM)**	**HeLa**	**U87**	**HEK293**
A-kinase anchor protein 13	AKAP13	3.1	3.2	4.3	0.0	0.0
Antigen peptide transporter 2	TAP2	2.8	2.8	3.0	−0.6	−0.6
DBIRD complex subunit ZNF326 ^A, BH^	ZNF326	2.9	3.2	5.0	0.0	0.0
E3 ubiquitin-protein ligase UBR4 ^BH^	UBR4	2.8	3.0	6.5	0.0	0.0
G patch domain and KOW motifs-containing protein	GPKOW	2.7	2.7	5.2	0.0	0.0
Intercellular adhesion molecule 1 ^A, BH^	ICAM1	3.6	3.6	4.4	0.0	0.0
Leucine-rich repeat-containing protein 16A	LRRC16A	2.7	2.6	4.0	0.0	0.0
Melanoma-associated antigen D2	MAGED2	2.8	2.5	4.4	0.0	0.0
Periostin	POSTN	3.2	2.6	2.6	−1.4	−1.4
Phospholipase A-2-activating protein ^B, BH^	PLAA	3.2	2.8	5.1	−1.1	−1.1
Protein RCC2 ^BH^	RCC2	2.8	2.7	3.8	−2.1	−2.1
Protein S100-P ^A, B, BH^	S100P	2.9	2.6	3.9	0.0	0.0
Receptor-type tyrosine-protein phosphatase F ^BH^	PTPRF	3.3	3.0	2.7	−2.3	−2.3
RNA-binding protein 10 ^BH^	RBM10	3.0	2.5	4.2	−0.6	−0.6
Shootin-1	KIAA1598	2.6	2.6	4.5	−0.6	−0.6
Sterile alpha motif domain-containing protein 9 ^A^	SAMD9	3.8	3.9	2.8	0.0	0.0
Thrombospondin-1	THBS1	3.5	3.1	6.1	0.0	0.0
Ubiquitin-like protein ISG15	ISG15	2.5	3.4	3.4	0.0	0.0
Zinc finger RNA-binding protein ^BH^	ZFR	2.7	3.7	5.8	0.0	0.0
**B.**	**Gene name**	**EC (LCM)**	**LC (LCM)**	**HeLa**	**U87**	**HEK293**
Calcium-activated chloride channel regulator 4	CLCA4	2.8	3.3	0.0	0.0	0.0
Carcinoembryonic antigen-related cell adhesion molecule 5 ^A, BH^	CEACAM5	3.8	2.8	0.0	0.0	0.0
Cathelicidin antimicrobial peptide	CAMP	3.8	2.7	0.0	0.0	0.0
Collagen alpha-1(XII) chain	COL12A1	4.0	3.5	−2.1	−2.1	−2.1
Dimethylaniline monooxygenase [N-oxide-forming] 3	FMO3	3.5	2.6	0.0	0.0	0.0
E3 ubiquitin-protein ligase DTX3L ^A, BH^	DTX3L	3.7	3.2	0.0	0.0	0.0
Epithelial cell adhesion molecule	EPCAM	3.2	3.2	0.0	0.0	0.0
Fibulin-2	FBLN2	3.1	3.0	0.0	0.0	0.0
HLA class II histocompatibility antigen, DRB1-16 beta chain ^A, BH^	HLA-DRB1	3.7	3.3	−0.6	−0.6	−0.6
HLA class II histocompatibility antigen, DRB1-7 beta chain	HLA-DRB1	3.8	3.0	0.0	0.0	0.0
Myeloperoxidase ^A^	MPO	5.0	3.6	0.0	0.0	0.0
Poly [ADP-ribose] polymerase 9 ^A, BH^	PARP9	3.7	2.6	0.0	0.0	0.0
Stimulator of interferon genes protein	TMEM173	3.0	2.8	0.0	0.0	0.0
Transgelin	TAGLN	3.5	2.9	−1.7	−1.7	−1.7

Panel A represents nineteen up-regulated proteins found in the early and late stage cervical cancer of LCM-derived cells and HeLa only. Panel B shows fourteen significant proteins exclusively found by LCM. For both panels, the following criterion was used: ^2^log fold-change ≥ 2.5, zero counts were converted to 0.125 to enable log calculations. Proteins indicated with (superscripts) ^A^, ^B^ and ^BH^ were found by the “all-or-nothing”, Bonferroni and Benjamini-Hochberg correction for multiple testing, respectively.

EC: early stage cervical cancer.

LC: late stage cervical cancer.

### Quantification of MCM3, CEACAM5, S100P and ICAM1 in whole tissue lysates by PRM

**Figure 8:**
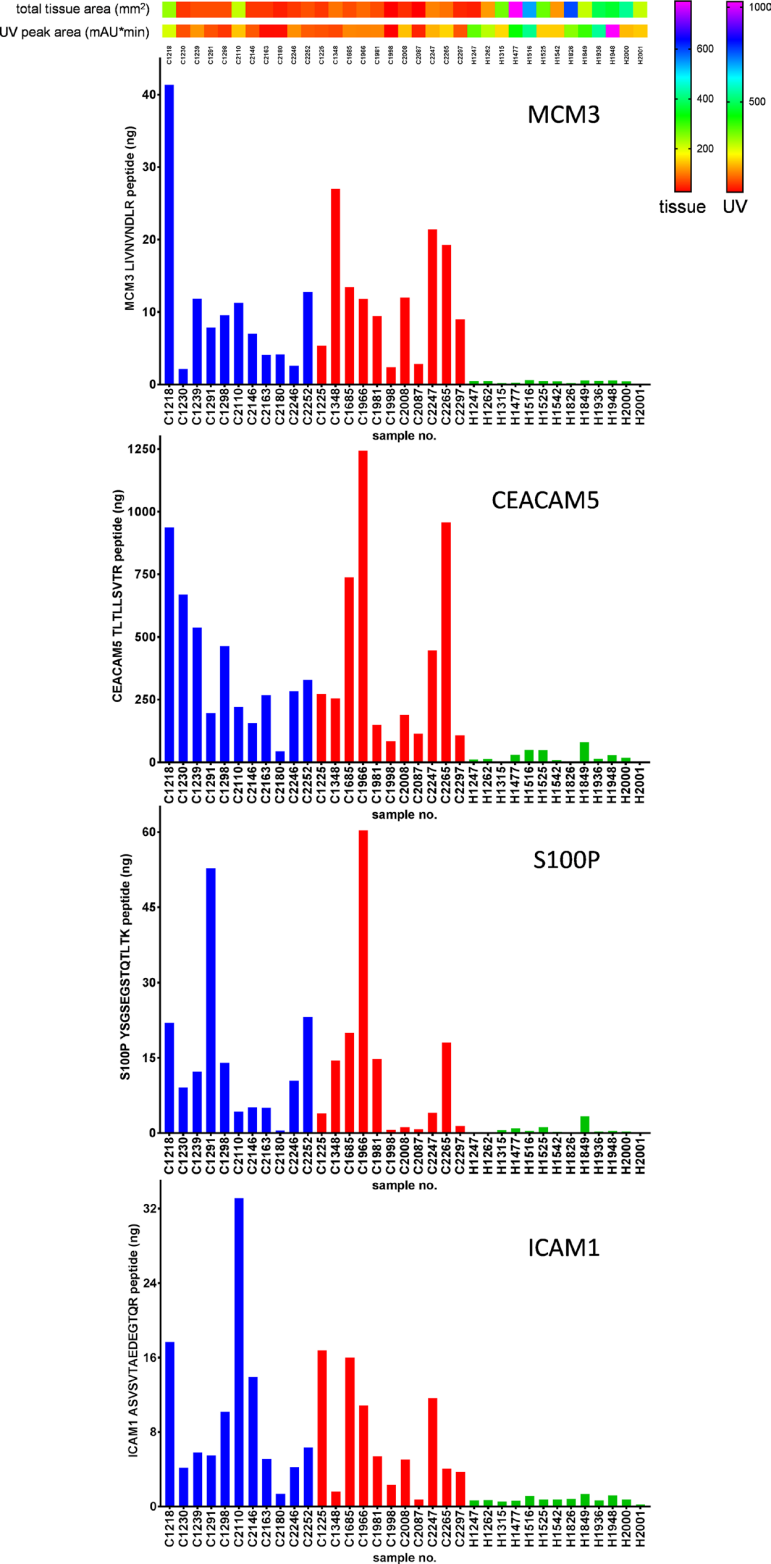
Example of targeted mass spectrometry by PRM Total amount (in nanograms) of the proteins *MCM3*, *CEACAM5*, *S100P* and *ICAM1* were determined in digests of whole tissue lysates. Total tissue areas (mm^2^) and UV peak areas (mAU*min) were applied on top of the figure to estimate the amount of tissue used. Blue, red and green bars correspond to early stage cancer, late stage cancer and healthy samples, respectively.

The concentrations of four proteins, *MCM3*, *CEACAM5*, *S100P* and *ICAM1*, that were highly significant in cervical cancer tissue found by the shotgun approach of each sample are represented in Supplementary Excel Data File 1. The PRM data have been deposited to the ProteomeXchange Consortium via the PRIDE [25] partner repository with the dataset identifier PXD008723 and 10.6019/PXD008723. An example of the calculated concentrations of the four targeted proteins by PRM is illustrated in Figure 8. Linearity, LOD (limit of detection), LOQ (limit of quantification) and reproducibility of serial dilutions of SIL peptide standards are represented in Supplementary Excel Data File 2.

### Comparison of protein profiles with transcriptome data

All proteins of the “all-or-nothing” and Bonferroni analyses (*n* = 55, which were commonly and exclusively found for both analyses, see Table 2A and 2B, respectively) were observed as relative high gene expression levels extracted from transcriptome data (Ojesina *et al.* [10]). Most of these overlapping proteins were found as highly differential in early stage cervical cancer when compared to the average gene expression level in the Ojesina *et al.* transcriptome data. This is illustrated in the Supplementary Figure 1 (“all-or-nothing” analysis) and Supplementary Figure 2A (Bonferroni analysis). In these heat maps it is shown that only four out of the 55 selected genes have low abundance, i.e. *MPO* (Supplementary Figure 1), *CRNN*, *DSG1*, and *PDIA3* (Supplementary Figure 2A); *CRNN* and *DSG* in agreement with our proteomics study. The other 51 out of 55 genes were expressed at relatively high level, around ^2^log (FPKM) of 5. In general, many genes had undetectable transcript expressions, with ^2^log (FPKM) below −5 and those which were expressed had low values, i.e. ^2^log (FPKM) of 3 and 4. To prove that genes were differentially expressed on protein level among highly expressed genes on RNA level, an analysis of gene expression of a background gene list was performed. This set was based on a selection of genes, corresponding to the list of the total identified proteins from LCM-derived samples (where 3,847 out of 4,138 genes from single peptide identification with removal of decoys were found in the transcriptome sequencing data) showed that about 10% of the proteins were expressed at a very low level, see Supplementary Figure 2B. With this taken in account, for the early stage cervical cancer analysis using Bonferroni (Table 2B) and for both early (Supplementary Table 2) and late stage cervical cancer analysis (Supplementary Table 3) using Benjamini-Hochberg the genes were significantly higher expressed (compared to background set, Wilcoxon rank-sum test, *p* < 0.01) on transcriptome level. The early stage cervical cancer using the “all-or-nothing” analysis (Table 2A) and the late stage cervical cancer using the Bonferroni analysis (Supplementary Table 1) did not exhibit a significantly elevated RNA expression (*p* = 0.427 and *p* = 0.395, respectively). Heat maps, which were created for proteins analyzed by Benjamini-Hochberg for the comparison of individual early and late stage cervical cancer with healthy epithelium are represented in the Supplementary Figures 3 and 4, respectively.

Furthermore, mutated or expressed genes as listed by Ojesina *et al.* were compared for presence or absence in the total number of 2,989 proteins (identified with at least one peptide) identified by our LCM approach. The comparisons are compiled in Supplementary Table 4 resulting in five overlapping proteins (*CBFB*, *CEACAM5*, *MAPK1*, *PARN* and *TP63*). For this panel, only *CEACAM5* was significantly different in abundance (*p* = 0.007) between early stage cervical cancer and healthy epithelium found by LCM and mass spectrometry.

## DISCUSSION

The combination of laser capture microdissection and high-resolution mass spectrometry in tissue sections from squamous cell cervical cancer patients showed increased levels of proteins in tumor tissue versus healthy epithelium and stroma. Most striking was the increase in the members of the MCM family (up to 32-fold increase in abundance) and associated proteins. MCM proteins are molecular motors that unwind duplex DNA and power fork progression during DNA replication. This process starts with initiation of DNA-replication occurring during S phase when two copies of each chromosome are present. *Cdc6* and *Cdt1* are then recruited by the origins recognition complex (ORC) and they in turn recruit the MCM complex to the ORC, forming the pre-RC and licensing the DNA for replication [26–28]. Because of its critical role during DNA replication, deregulation of MCM function contributes to human carcinogenesis [29]. Previous studies have shown that MCM proteins are highly expressed in various malignant human cancers and in cells at an early stage of malignant transformation [30, 31]. Members of the MCM family have also been described as diagnostic cancer markers, because they are not expressed in quiescent somatic cells that have been arrested in the G_0_ phase of the cell cycle. We and others hypothesize that up-regulation of MCM proteins is critical for tumor progression and that MCM proteins might serve as viable targets for anti-cancer therapy as well as molecular markers for diagnosis.

As mentioned above, we used three analysis techniques that were restrictive in finding differential proteins. In addition, we used an extra threshold by a permutation approach in the protein network finding. Using these restrictive analyses, we believe to be confident in finding the most discriminative proteins and networks of these proteins. Apparently, the differences between cervical cancer cells and healthy epithelial cells were that large that we were able to find relatively high number of proteins that fulfill these restrictions.

Our IPA analysis indicated that the biological interactions of the 30 proteins identified by Bonferroni related to cervical cancer correlated to the network “DNA Replication, Recombination, and Repair”. The same network was assigned to analyses determined by Benjamini-Hochberg. Remarkably, another network “Cardiac Arrhythmia, Cardiovascular Disease, Organismal Injury and Abnormalities” that was found with the proteins from the Benjamini-Hochberg analysis was filled with a high number of down-regulated proteins. Most probably cervical specific proteins are lost in early cancer cells during the tumor oncogenesis. For diagnostic purposes, elevation of a protein marker could be more applicable than down-regulated proteins.

We found nineteen proteins (Table 4) that were up-regulated in cervical cancer (early and late stage) tissue and HeLa when compared to healthy epithelium from controls obtained by LCM. Some of these proteins might be cervical cancer-related, since they were not up-regulated in cells from other non-cervical HEK and U87 cell cultures but only in the HeLa cell line. Nevertheless, more cervical cancer cell lines ought to be investigated to confirm this. One of these proteins is *CEACAM5*, a protein with HPV integration sites that was also found by Ojesina *et al.* [10] to be highly expressed (Supplementary Figure 1). CEACAM5 is related to cell-adhesion molecules and belongs to the carcinoembryonic antigen (CEA) gene family. It is strongly expressed in epithelial cells and known as a tumor marker for early detection of recurrent disease due to its expression in several adenocarcinomas (e.g. colon, lung, breast, ovarian) [32], however, there is no literature related to cervical cancer.

The coding mutation in *ERBB2* described by Ojesina *et al.* [10] as being specific for cervical cancer could not be detected by our LCM approach. *MAPK1*, also described *as being* specific for cervical cancer was detected. However, the abundance level did not change in cervical cancer compared to healthy epithelial or stroma cells according to our analysis. *S100P* was found to be up-regulated in cervical cancer tissue from early and late stage patients as well as in HeLa cells. It was not found in healthy epithelium, stroma, U87 and HEK293 cells, although it is overexpressed in several other cancers [33]. *S100P* is a member of the S100 protein family that is characterized by its calcium-binding properties due to structural motifs containing 2 EF-hand domains. *S100P* was first isolated from human placenta and has a crucial role in several biological functions; however, its exact function in cervical cancer remains unclear [33, 34]. Quantitative proteomics by PRM was used to confirm the observation of four proteins. It was shown that full agreement was obtained on digests of whole tissue lysates. As sensitivity is in PRM much better than in shotgun proteomics, an almost all-or-nothing difference was observed between the cancer and healthy samples. The difference for *MCM3* was detectable for all samples with roughly two to three orders of magnitude.

National population-based cervical cancer screening programs have reduced the incidence of cervical cancer significantly in the western world [35]. However, in both the technical aspects and performance of different screening test there is room for improvement. The most widely used cervical cancer screening test is cytology-based testing. Primary screening for cervical cancer is currently changing in many countries including the Netherlands. HrHPV testing is becoming the preferred primary screening test over cytology. By hrHPV testing the sensitivity for detecting premalignant lesions is much higher [36–38]. Because of this increased sensitivity, more CIN2+ lesions will be detected and less carcinomas will be missed. A disadvantage of the hrHPV test for primary screening is the lower specificity of this test, resulting from detection of women with transient HPV infection who will not develop (premalignant) cervical cancer. To prevent unnecessary referral to the gynecologist and associated high costs, there is a need for risk stratification by triage testing of hrHPV positive women and the four selected proteins for PRM might help in that respect.

Simple, more specific biomarkers for cervical cancer and its precancerous stages are required that can be used on e.g. liquid-based cytology (LBC) samples. These samples used for routine screening in a precancerous stage could be used for targeting differentially expressed proteins in a multiplexed manner (e.g. >50 proteins). LBC samples can be related much more directly to cervical tissue because they are less complex compared to e.g. sera where the extreme range of different serum proteins puts limits on the detection of proteins in the ng/mL range without the use of antibody or other new affinity enrichment steps.

In conclusion, we have shown that LCM proteomics with high-resolution mass spectrometry is a viable approach to detect differences in individual protein levels in tissues of early stage squamous cell cervical cancer patients compared to healthy control tissue. For four proteins we were able to confirm in a quantitative way these rather large differences in protein levels. In addition, we have found two significant differential networks. A down-regulated network that probably shows the loss of cervical specific proteins in cancer cells and a highly-up-regulated network that relates to a cancer mechanism that involves proliferation and progression in cervical cancer. These networks can be used as sources for diagnostics and will add to understanding the early processes in cervical cancer.

## MATERIALS AND METHODS

### Sample procurement

All patients referred to the outpatient clinic of the University Medical Center Groningen (UMCG) with squamous cervical cancer were routinely asked to participate in our ongoing ‘Methylation study’ which has been approved by the Institutional Review Board (IRB) of the UMCG. Cervical tissue and clinicopathologic data were prospectively collected and stored in our tissue bank. Within our ‘Methylation study’ tissue samples and clinicopathologic data that were collected from normal cervical tissues, were also collected from patients who planned to undergo a hysterectomy for non-malignant reasons. All cervical tissues that were used for the healthy control group was judged as histopathological normal. For determination of the percentage of tumor in the cancer specimen, 4 μm sections were cut and haematoxylin & eosin (HE) stained. Only specimens with more than 70% tumor were used. For LCM, we selected frozen tissue of 22 SCC patients and from thirteen controls (Table 1). The median age of the cervical cancer patients was 55 years (IQR 44–75) and for the patients with normal cervices 43 years (IQR 41–50). The stage of SCC patients was: 3 (14%) FIGO stage IB1, 5 (23%) FIGO stage IB2, 3 (14%) FIGO stage IIB, 2 (9%) FIGO stage IIIA, 6 (27%) FIGO stage IIIB and 3 (14%) FIGO stage IV. FIGO stages I–II and III–IV were defined as early stage and late stage cervical cancer, respectively. All tissues were primary tumors and patients did not receive neoadjuvant therapy.

### LCM

Areas for LCM were selected on HE-stained cryosections. Cryosections of 10 μm were prepared from each of the eleven early and eleven late stage cervical cancer, and the thirteen healthy cervical tissue samples. The sections were mounted on polyethylene naphthalate (PEN)-covered glass slides (Carl Zeiss, Zwijndrecht, the Netherlands). An area corresponding to approximately 8,000 cells was microdissected. We calculated the number of cells by assuming an average volume of 10 × 10 × 10 μm for one cell. The tissue pieces were collected in 20 μL 0.1% Rapigest SF detergent (Waters, Milford, MA, USA) and reduced using 4.7 mM dithiothreitol at 60° C for 30 minutes followed by alkylation using 16.9 mM iodoacetamide in the dark at room temperature for 30 minutes. Subsequently, the tissue pieces were enzymatically digested by adding 2 μL trypsin (Mass Spectrometry grade, 100 μg/mL in 3 mM Tris-HCl, pH 8.8) at 37° C overnight.

### Protein identification by nano-LC-MS/MS

Online nano-LC-ESI-MS/MS using an application of a shotgun proteomics workflow was used for identification of tryptic peptides. A digested peptide mixture equivalent to 8,000 cells was separated on an Ultimate 3000 HPLC system (Thermo Fisher Scientific, Germering, Germany) and subsequently online measured in an Orbitrap Fusion mass spectrometer (Thermo Fisher Scientific, San Jose, CA, USA) in the data-dependent acquisition mode. Samples were loaded on to a trap column (PepMap C18, 300 μm ID, 5 mm length, 5 μm particle size, 100 Å pore size; Thermo Fisher Scientific), washed and desalted for 8 minutes using 0.1% trifluoroacetic acid as loading solvent at a flow rate of 20 μL/min. The trap column was switched in-line with the analytical column (PepMap C18, 75 μm ID × 500 mm, 2 μm particle size and 100 Å pore size, Thermo Fisher Scientific) and peptides were eluted with a 90-minute acetonitrile gradient ranging from 3% to 30% (and formic acid concentration from 0.1% to 0.08%, respectively). All LC solvents were purchased at Biosolve (Valkenswaard, the Netherlands). Column flow rate was set to 250 nL/min, and eluting peptides were measured at 214 nm in a 3 nL nano flow cell (Thermo Fisher Scientific), online coupled to the mass spectrometer. For electrospray ionization nano ESI emitters (New Objective, Woburn, MA, USA) were used and a spray voltage of 1.7 kV was applied. For MS detection, a data-dependent acquisition method was used with a survey scan from 350–1650 Th at 120,000 resolution (AGC target 400,000) and consecutively isolated and fragmented by collisional induced dissociation (CID) at 35% normalized collision energy (AGC target 10,000) of the most abundant precursors in the linear ion trap until a duty cycle time of 3 seconds was reached (‘Top Speed’ method). Precursor masses that were selected once for MS/MS were excluded from further fragmentation for the next 60 seconds.

Proteins from the LCM-derived samples were assigned by exporting features, for which MS/MS spectra were recorded, using the ProteoWizard software (version 3.0.9248; http://proteowizard.sourceforge.net). Resulting .mgf files were submitted to Mascot (version 2.3.01, Matrix Science, London, UK) and applied to the human database (UniProtKB/Swiss-Prot, version 2013_07, human taxonomy, 20,265 entries) for protein identifications assuming trypsin digestion and applying the following parameters: fragment ion mass tolerance of 0.50 Da, parent ion mass tolerance of 10 ppm, maximum number of missed cleavages of two. Oxidation of methionine was specified in Mascot as a variable modification, while carbamidomethylation of cysteine was set as a fixed modification. Scaffold (version 4.7.2, Proteome Software Inc., Portland, OR) was used to summarize and filter the MS/MS-based peptide data from all Mascot searches. The number of proteins was derived from the peptide data according to the following criteria. Peptide identifications needed to have more than 95% probability as specified by the Peptide Prophet algorithm. Protein identifications had to have more than 99% probability and contain at least one peptide identified.

### Data analysis

**Figure 9:**
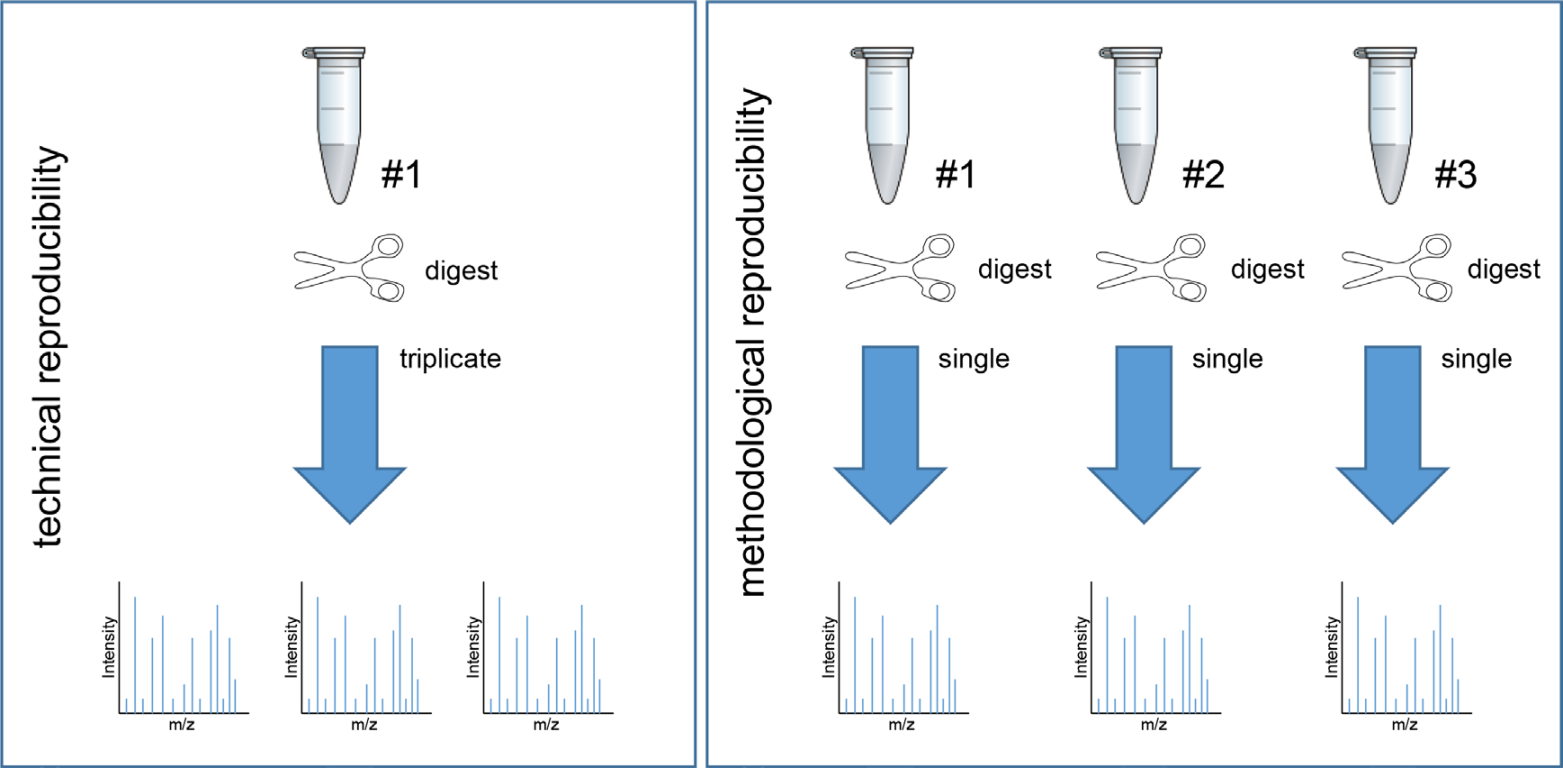
Experimental design of technical and methodological reproducibility

Technical and methodological reproducibility was determined by performing the measurements in triplicate and doing the entire experiment (microdissection and tryptic digestion) also in triplicate. For technical reproducibility, a tryptic digested tissue lysate was used, while for methodological reproducibility an LCM-derived sample was taken and measured. A diagram illustrating the experimental design is shown in Figure 9. For both reproducibility types, the total number of overlapping identified proteins was analyzed by Scaffold and eventually the percentage was used as a readout. For both reproducibility types an overlap greater than 50% was indicated as acceptable [39, 40].

A volcano plot was created to determine changes in protein abundances indicated as ^2^log fold-changes between individual early and late stage cervical cancer with the healthy epithelium group (*p* < 0.05). The ^2^log fold-changes were calculated based on comparison of the individual early and late stage cervical cancer with healthy epithelium according to spectral counts (zero counts were converted to 0.125 to enable log calculations). Three different analysis methods were chosen for comparison of the early stage cervical cancer with the healthy epithelium and late stage cervical cancer group with the healthy epithelium group. Two stringent analyses were used i.e. an analysis based on an “all-or-nothing” criteria and second a Bonferroni analysis to correct for multiple testing, and third a less stringent correction for multiple testing i.e. Benjamini-Hochberg. The “all-or-nothing” analysis was deemed discriminative when 1) a protein was not identified in healthy epithelium and stroma and when 2) a protein was at least identified in seven out of the eleven early stage cervical cancer subjects. In addition, the identified proteins from early stage cervical cancer were allowed to be present in late stage cervical cancer. We have chosen for these three analyses to classify in a restrictive way (Bonferroni) and a more relaxed analysis (Benjamini-Hochberg), and the “all-or-nothing” analysis that offers more possibilities to handle missing values in mass spectrometry. The differentially abundant proteins found by these three analysis methods, have been introduced into pathway analysis using the IPA software [41]. Data were entered into the IPA tool to assign the differentially expressed proteins to different network interactions according to their respective significance levels to fit in that network. A permutation test was performed to determine a threshold score by selection of random 31 (comparable numbers with those analyzed by the “all-or-nothing” criteria), 30 and 13 proteins (comparable numbers with those analyzed by Bonferroni analysis between individual early and late stage cervical cancer with healthy epithelium, respectively), 319 and 140 proteins (comparable numbers with those analyzed by Benjamini-Hochberg between individual early and late stage cervical cancer with healthy epithelium, respectively) that were extracted from the UniProt database (uniprot_sprot_HUMAN_v151112; 20194 entries). The test was repeated ten times and from the networks with highest score (as calculated by IPA using right-tailed Fisher's Exact test) the mean value of the ten repeats was calculated and subsequently the lower- and upper 95% confidence level (Cl) of the mean were calculated. Network scores which exceeded the 95% Cl of the mean threshold (assumed as background) were taken into account as confidential differential.

To determine the heterogeneity among patients, a hierarchical clustering analysis was performed of fourteen differential IPA classified proteins that belonged to Bonferroni analysis when comparing early stage cervical cancer with healthy epithelium. The IPA classified proteins were clustered individually in samples from the groups early and late stage cervical cancer and healthy epithelium. For hierarchical clustering analysis PermutMatrix 1.9.3. was used (http://www.atgc-montpellier.fr/permutmatrix) selecting the Euclidean distance for dissimilarity, Wards’ method, as a cluster method and Bipolarization seriation [42, 43].

### Confirmation by PRM mass spectrometry

Identical numbers of the tissue samples (Table 1) were measured by PRM to quantify *MCM3*, *CEACAM5*, *S100P* and *ICAM1* in digests of whole tissue lysates using stable isotope-labeled (SIL) peptides purchased from Pepscan BV (Lelystad, the Netherlands). A subset of nine peptides were selected to be quantified by PRM (Supplementary Table 5). Collected tissue pieces were compiled in 200 μL 0.1% Rapigest SF detergent, sonicated for 3 minutes using a horn sonifier bath (Ultrasonic Disruptor Sonifier II, Bransons Utrasonics, Danbury, CT, USA) at 85% amplitude and heated for 5 minutes at 99° C for protein denaturation. Subsequently, 50 out of the 200 μL tissue lysates were each spiked with 10 fmol of the SIL peptides prior to enzymatic digestion. Samples were subsequently digested by adding 2 μg trypsin at 37° C overnight. One microliter of each sample was measured by PRM, performed on an Orbitrap Fusion instrument according to our previous work [44]. Analytical parameters such as linearity, LOD and LOQ were determined. For reproducibility, CVs were calculated for each serial dilution (0, 0.625, 1.25, 2,5, 5, 10 and 20 fmol/uL) using triplicate measurements.

### Comparison of protein profiles in LCM-derived early stage cervical cancer tissue with HeLa, U87 and HEK293 cell lines

The identified proteins from the “all-or-nothing”, Bonferroni and Benjamini-Hochberg analysis (comparison of the early stage cervical cancer tissue with healthy epithelium group only) were compared with proteins identified in the cervical cancer derived HeLa cell line and two other cell lines that are unrelated to cervical cancer (U87; primary glioblastoma and HEK293; Human Embryonic Kidney cell line) by calculating ^2^log fold-changes related to LCM-derived healthy cervical epithelium. Proteins were filtered with the following criterion for each individual group: ^2^log fold-change relative to healthy epithelium > 1. For HeLa, a standard protein digest (catalog no. 88328, Thermo Scientific, Landsmeer, the Netherlands) was used. From U87 and HEK293 cell lines 10^6^ cells of each cell type were digested according to a protocol as recommended by Thermo Scientific (catalog no. 90110). For all three cell lines one μg of each digest was analyzed by nano-LC-Orbitrap MS/MS. The IPA classified proteins were compared with their abundances in the two cervical cancer groups (early and late stage) and in the various cell lines. Scaffold perSPECtives viewer (version 2.1.0, Proteome Software Inc., Portland, OR) was used for visualization of proteins found in cervical-derived tissue by using LCM simultaneously with results obtained from these three cell lines.

### Comparison to existing genomic data

Proteomic data were compared to transcriptome data published by Ojesina *et al.* [10]. Transformed ^2^log expression values were extracted from the transcriptome sequencing data of tumor samples consisting of squamous cell carcinoma and adenocarcinoma. These genomic data contained no expression data information about healthy tissue as indicated by Ojesina *et al.* and therefore no comparisons could be made to the healthy controls in our study. Heat maps were created of the proteins from the three analyses performed in this study (Table 2A, 2B; Supplementary Tables 1–3). Genes were selected only from the genomic analysis that overlapped with genes identified with the proteomic approach (set of 4,138 proteins with the removal of decoys). For this selection of genes, an average expression level (across all samples) was defined. Using Wilcoxon rank-sum test, the average mRNA expression levels of genes identified as differentially expressed in proteome analysis (represented in heat maps) was evaluated if this was different from the average mRNA expression products of other genes that were identified on protein level (Significance level *p* < 0.01).

## SUPPLEMENTARY MATERIALS FIGURES AND TABLES











## Abbreviations

CIN: Cervical intraepithelial neoplasia
FIGO: International Federation of Gynecology and Obstetrics
FPKM: Fragments Per Kilobase Million
HEK293: Human Embryonic Kidney 293 (cell line)
HPV: Human papillomavirus
IPA: Ingenuity Pathway Analysis
LCM: Laser capture microdissection
LOD: Limit of detection
LOQ: Limit of quantification
MCM: Minichromosome Maintenance
PRM: Parallel Reaction Monitoring
SCC: Squamous cell cervical cancer
U87: Uppsala 87 Malignant Glioma (cell line)

## References

[1] Fitzmaurice C, Dicker D, Pain A, Hamavid H, Moradi-Lakeh M, MacIntyre MF, Allen C, Hansen G, Woodbrook R, Wolfe C, Hamadeh RR, Moore A, Werdecker A (2013). Global Burden of Disease Cancer Collaboration. The Global Burden of Cancer.

[2] Franco EL, Schlecht NF, Saslow D (2003). The epidemiology of cervical cancer. Cancer journal.

[3] Soerjomataram I, Lortet-Tieulent J, Parkin DM, Ferlay J, Mathers C, Forman D, Bray F (2012). Global burden of cancer in 2008: a systematic analysis of disability-adjusted life-years in 12 world regions. Lancet.

[4] Parkin DM, Bray F, Ferlay J, Pisani P (2005). Global cancer statistics, 2002. CA Cancer J Clin.

[5] Cotton SC, Sharp L, Seth R, Masson LF, Little J, Cruickshank ME, Neal K, Waugh N, Group T (2007). Lifestyle and socio-demographic factors associated with high-risk HPV infection in UK women. Br J Cancer.

[6] Monsonego J (2004). EUROGIN. HPV infections and cervical cancer prevention. Priorities and new directions. Gynecol Oncol.

[7] Clifford GM, Smith JS, Aguado T, Franceschi S (2003). Comparison of HPV type distribution in high-grade cervical lesions and cervical cancer: a meta-analysis. Br J Cancer.

[8] Nguyen HH, Broker TR, Chow LT, Alvarez RD, Vu HL, Andrasi J, Brewer LR, Jin G, Mestecky J (2005). Immune responses to human papillomavirus in genital tract of women with cervical cancer. Gynecologic Oncology.

[9] Mirabello L, Yeager M, Yu K, Clifford GM, Xiao Y, Zhu B, Cullen M, Boland JF, Wentzensen N, Nelson CW, Raine-Bennett T, Chen Z, Bass S (2017). HPV16 E7 Genetic Conservation Is Critical to Carcinogenesis. Cell.

[10] Ojesina AI, Lichtenstein L, Freeman SS, Pedamallu CS, Imaz-Rosshandler I, Pugh TJ, Cherniack AD, Ambrogio L, Cibulskis K, Bertelsen B, Romero-Cordoba S, Trevino V, Vazquez-Santillan K (2014). Landscape of genomic alterations in cervical carcinomas. Nature.

[11] Cancer Genome Atlas Research Network, Albert Einstein College of Medicine, Analytical Biological Services, Barretos Cancer Hospital, Baylor College of Medicine, Beckman Research Institute of City of Hope, Buck Institute for Research on Aging, Canada's Michael Smith Genome Sciences Centre, Harvard Medical School, Helen F. Graham Cancer Center & Research Institute at Christiana Care Health Services, HudsonAlpha Institute for Biotechnology, ILSbio, LLC, Indiana University School of Medicine (2017). Integrated genomic and molecular characterization of cervical cancer. Nature.

[12] Zhang H, Liu T, Zhang Z, Payne SH, Zhang B, McDermott JE, Zhou JY, Petyuk VA, Chen L, Ray D, Sun S, Yang F, Chen L (2016). Integrated Proteogenomic Characterization of Human High-Grade Serous Ovarian Cancer. Cell.

[13] Mertins P, Mani DR, Ruggles KV, Gillette MA, Clauser KR, Wang P, Wang X, Qiao JW, Cao S, Petralia F, Kawaler E, Mundt F, Krug K (2016). Proteogenomics connects somatic mutations to signalling in breast cancer. Nature.

[14] Datta S, Malhotra L, Dickerson R, Chaffee S, Sen CK, Roy S (2015). Laser capture microdissection: Big data from small samples. Histol Histopathol.

[15] Langenkamp E, Kamps JA, Mrug M, Verpoorte E, Niyaz Y, Horvatovich P, Bischoff R, Struijker-Boudier H, Molema G (2013). Innovations in studying *in vivo* cell behavior and pharmacology in complex tissues—microvascular endothelial cells in the spotlight. Cell Tissue Res.

[16] Liu NQ, Braakman RB, Stingl C, Luider TM, Martens JW, Foekens JA, Umar A (2012). Proteomics pipeline for biomarker discovery of laser capture microdissected breast cancer tissue. J Mammary Gland Biol Neoplasia.

[17] Okayama A, Miyagi Y, Oshita F, Nishi M, Nakamura Y, Nagashima Y, Akimoto K, Ryo A, Hirano H (2014). Proteomic analysis of proteins related to prognosis of lung adenocarcinoma. J Proteome Res.

[18] Liu NQ, Dekker LJ, Stingl C, Guzel C, De Marchi T, Martens JW, Foekens JA, Luider TM, Umar A (2013). Quantitative proteomic analysis of microdissected breast cancer tissues: comparison of label-free and SILAC-based quantification with shotgun, directed, and targeted MS approaches. J Proteome Res.

[19] De Marchi T, Liu NQ, Stingl C, Timmermans MA, Smid M, Look MP, Tjoa M, Braakman RB, Opdam M, Linn SC, Sweep FC, Span PN, Kliffen M (2015). 4-protein signature predicting tamoxifen treatment outcome in recurrent breast cancer. Mol Oncol.

[20] Mu Y, Chen Y, Zhang G, Zhan X, Li Y, Liu T, Li G, Li M, Xiao Z, Gong X, Chen Z (2013). Identification of stromal differentially expressed proteins in the colon carcinoma by quantitative proteomics. Electrophoresis.

[21] Guzel C, Ursem NT, Dekker LJ, Derkx P, Joore J, van Dijk E, Ligtvoet G, Steegers EA, Luider TM (2011). Multiple reaction monitoring assay for pre-eclampsia related calcyclin peptides in formalin fixed paraffin embedded placenta. J Proteome Res.

[22] Gu Y, Wu SL, Meyer JL, Hancock WS, Burg LJ, Linder J, Hanlon DW, Karger BL (2007). Proteomic Analysis of High-Grade Dysplastic Cervical Cells Obtained from ThinPrep Slides Using Laser Capture Microdissection and Mass Spectrometry. Journal of Proteome Research.

[23] Tsai FL, Vijayraghavan S, Prinz J, MacAlpine HK, MacAlpine DM, Schwacha A (2015). Mcm2–7 Is an Active Player in the DNA Replication Checkpoint Signaling Cascade via Proposed Modulation of Its DNA Gate. Molecular and cellular biology.

[24] Das M, Prasad SB, Yadav SS, Modi A, Singh S, Pradhan S, Narayan G (2015). HPV-type-specific response of cervical cancer cells to cisplatin after silencing replication licensing factor MCM4. Tumour Biol.

[25] Vizcaino JA, Csordas A, del-Toro N, Dianes JA, Griss J, Lavidas I, Mayer G, Perez-Riverol Y, Reisinger F, Ternent T, Xu QW, Wang R, Hermjakob H (2016). Update of the PRIDE database and its related tools. Nucleic Acids Res.

[26] Bochman ML, Schwacha A (2009). The Mcm complex: unwinding the mechanism of a replicative helicase. Microbiol Mol Biol Rev.

[27] Sanchez-Berrondo J, Mesa P, Ibarra A, Martinez-Jimenez MI, Blanco L, Mendez J, Boskovic J, Montoya G (2012). Molecular architecture of a multifunctional MCM complex. Nucleic Acids Res.

[28] Tye BK (1999). MCM proteins in DNA replication. Annu Rev Biochem.

[29] Honeycutt KA, Chen Z, Koster MI, Miers M, Nuchtern J, Hicks J, Roop DR, Shohet JM (2006). Deregulated minichromosomal maintenance protein MCM7 contributes to oncogene driven tumorigenesis. Oncogene.

[30] Lei M (2005). The MCM complex: its role in DNA replication and implications for cancer therapy. Curr Cancer Drug Targets.

[31] Majid S, Dar AA, Saini S, Chen Y, Shahryari V, Liu J, Zaman MS, Hirata H, Yamamura S, Ueno K, Tanaka Y, Dahiya R (2010). Regulation of minichromosome maintenance gene family by microRNA-1296 and genistein in prostate cancer. Cancer Res.

[32] Blumenthal RD, Leon E, Hansen HJ, Goldenberg DM (2007). Expression patterns of CEACAM5 and CEACAM6 in primary and metastatic cancers. BMC Cancer.

[33] Parkkila S, Pan PW, Ward A, Gibadulinova A, Oveckova I, Pastorekova S, Pastorek J, Martinez AR, Helin HO, Isola J (2008). The calcium-binding protein S100P in normal and malignant human tissues. BMC Clin Pathol.

[34] Arumugam T, Logsdon CD (2011). S100P: a novel therapeutic target for cancer. Amino Acids.

[35] van der Aa MA, Pukkala E, Coebergh JW, Anttila A, Siesling S (2008). Mass screening programmes and trends in cervical cancer in Finland and the Netherlands. Int J Cancer.

[36] Bulkmans NW, Rozendaal L, Snijders PJ, Voorhorst FJ, Boeke AJ, Zandwijken GR, van Kemenade FJ, Verheijen RH, v Groningen K, Boon ME, Keuning HJ, van Ballegooijen M, van den Brule AJ, Meijer CJ (2004). POBASCAM, a population-based randomized controlled trial for implementation of high-risk HPV testing in cervical screening: design, methods and baseline data of 44,102 women. Int J Cancer.

[37] Mayrand MH, Duarte-Franco E, Rodrigues I, Walter SD, Hanley J, Ferenczy A, Ratnam S, Coutlee F, Franco EL, Canadian Cervical Cancer Screening Trial Study Group (2007). Human papillomavirus DNA versus Papanicolaou screening tests for cervical cancer. N Engl J Med.

[38] Arbyn M, Ronco G, Anttila A, Meijer CJ, Poljak M, Ogilvie G, Koliopoulos G, Naucler P, Sankaranarayanan R, Peto J (2012). Evidence regarding human papillomavirus testing in secondary prevention of cervical cancer. Vaccine.

[39] Stingl C, van Vilsteren FG, Guzel C, Ten Kate FJ, Visser M, Krishnadath KK, Bergman JJ, Luider TM (2011). Reproducibility of protein identification of selected cell types in Barrett's esophagus analyzed by combining laser-capture microdissection and mass spectrometry. J Proteome Res.

[40] Tabb DL, Vega-Montoto L, Rudnick PA, Variyath AM, Ham AJ, Bunk DM, Kilpatrick LE, Billheimer DD, Blackman RK, Cardasis HL, Carr SA, Clauser KR, Jaffe JD (2010). Repeatability and reproducibility in proteomic identifications by liquid chromatography-tandem mass spectrometry. J Proteome Res.

[41] Kramer A, Green J, Pollard J, Tugendreich S (2014). Causal analysis approaches in Ingenuity Pathway Analysis. Bioinformatics.

[42] Meunier B, Dumas E, Piec I, Bechet D, Hebraud M, Hocquette JF (2007). Assessment of hierarchical clustering methodologies for proteomic data mining. J Proteome Res.

[43] Caraux G, Pinloche S (2005). PermutMatrix: a graphical environment to arrange gene expression profiles in optimal linear order. Bioinformatics.

[44] Güzel C, Govorukhina NI, Stingl C, Dekker LJM, Boichenko A, van der Zee AGJ, Bischoff RPH, Luider TM (2018). Comparison of Targeted Mass Spectrometry Techniques with an Immunoassay: A Case Study for HSP90alpha. Proteomics Clin Appl.

