# Supplementary material for: Proteomic alterations in early stage cervical cancer
Source: Oncotarget. 2018 Apr 6;9(26):18128–47. doi: 10.18632/oncotarget.24773 (PMC5915062; doi:10.18632/oncotarget.24773)
Supplement: Supplementary file 1 [file oncotarget-09-18128-s001.pdf]

Proteomic alterations in early stage cervical cancer

SUPPLEMENTARY MATERIALS

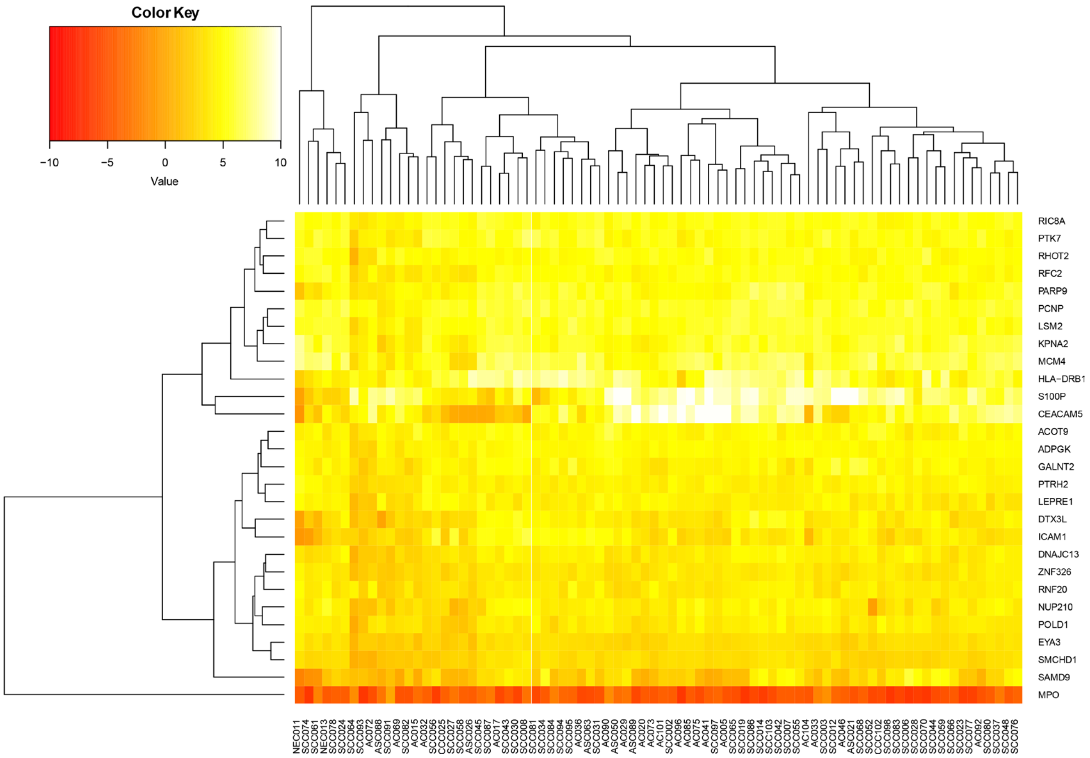

Supplementary Figure 1: Transcription level of genes identified by proteomics analysis. mRNA expression values were derived from Ojesina *et al*. dataset [10]. The significant proteins found by the "all-or-nothing" (n = 31, Table 2A) between early stage cervical cancer and healthy epithelium was not significantly correlated (Wilcoxon rank-sum test, $p = 0.427$) with ^{2}log transformed expression levels found in the genomics data published by Ojesina *et al*. Sample names indicate histology of tumor material: NEC - neuroendocrine carcinoma; SCC - squamous cell carcinoma; AC - adenocarcinoma; ASC - adenosquamous carcinoma; CCC - clear cell carcinoma.

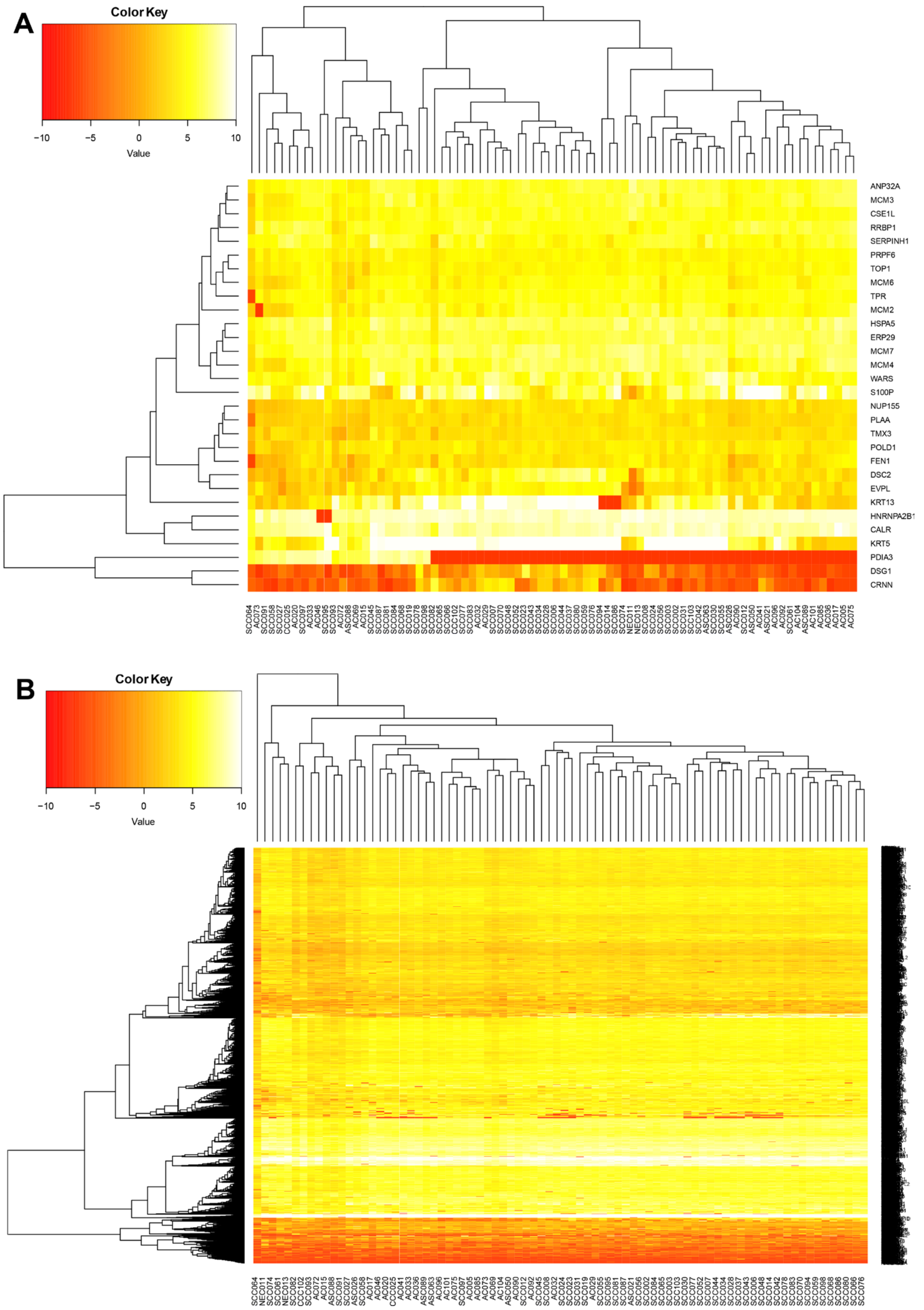

Supplementary Figure 2: Transcription level of genes identified by proteomics analysis. mRNA expression values were derived from Ojesina *et al*. dataset [10]. The significant proteins found after Bonferroni analysis (**A**) ($n = 30$, Table 2B) between early stage cervical cancer and healthy epithelium were significantly correlated (Wilcoxon rank-sum test, $p < 0.01$) with ^{2}log transformed expression levels found in the genomics data published. The background assessment based on all proteins identified by LCM that were correlated to the transcriptome sequencing data is shown in (**B**). Sample names indicate histology of tumor material: NEC - neuroendocrine carcinoma; SCC - squamous cell carcinoma; AC - adenocarcinoma; ASC - adenosquamous carcinoma; CCC - clear cell carcinoma.

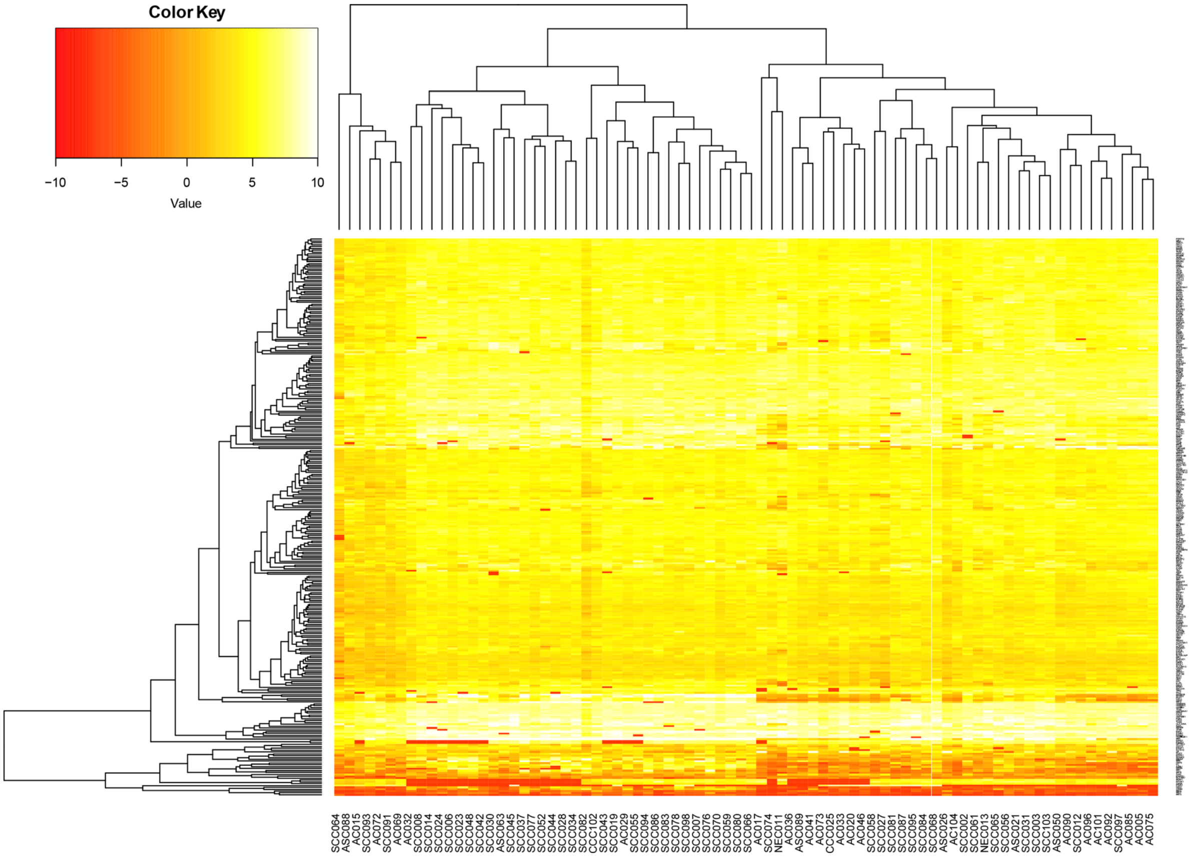

Supplementary Figure 3: Transcription level of genes identified by proteomics analysis. mRNA expression values were derived from Ojesina *et al*. dataset [10]. The significant proteins (n = 319; Supplementary Table 2) found by the Benjamini-Hochberg correction between early stage cervical cancer and healthy epithelium were significantly correlated (Wilcoxon rank-sum test $p = 3.12 \times 10–12$) with ^{2}log transformed expression levels found in the published transcriptomics dataset. Sample names indicate histology of tumor material: NEC - neuroendocrine carcinoma; SCC - squamous cell carcinoma; AC - adenocarcinoma; ASC - adenosquamous carcinoma; CCC - clear cell carcinoma.

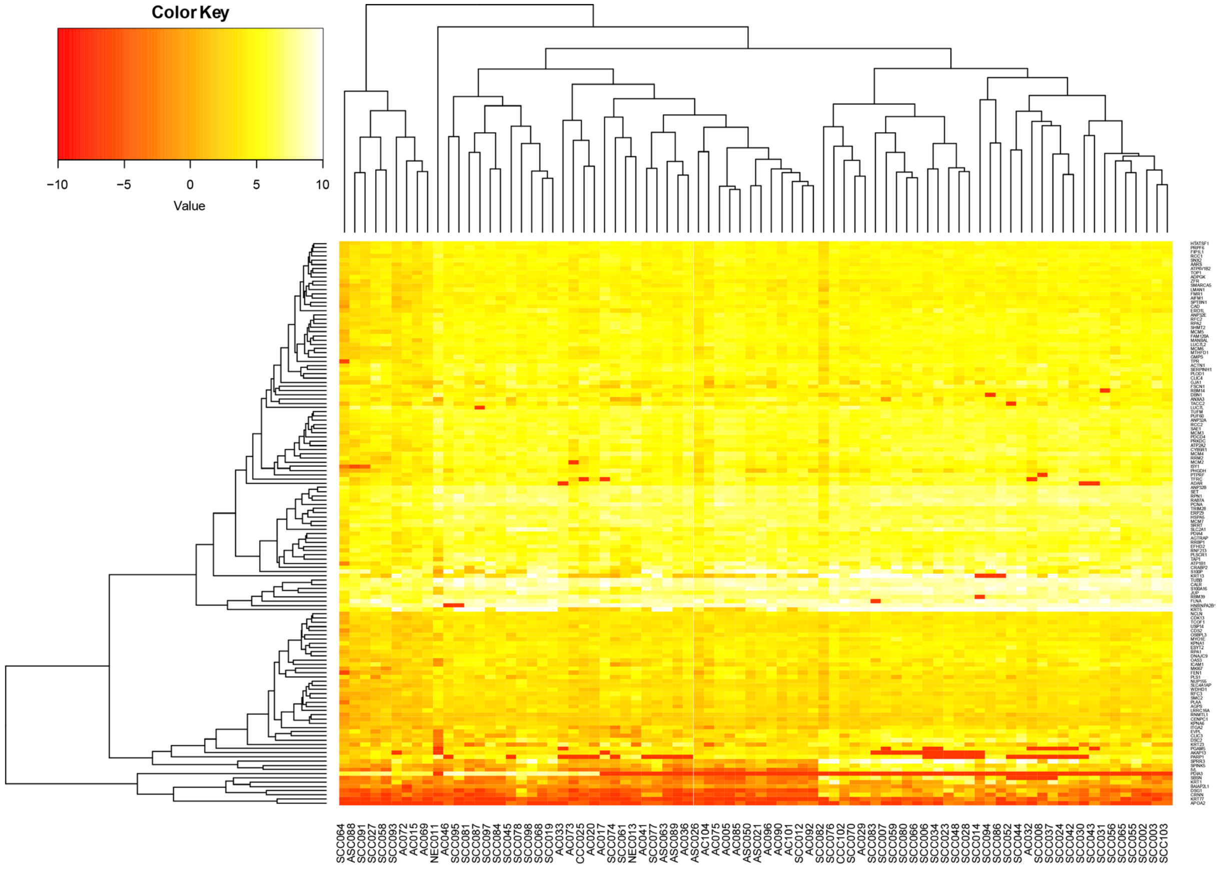

Supplementary Figure 4: Transcription level of genes identified by proteomics analysis. mRNA expression values were derived from Ojesina *et al*. dataset [10]. The significant proteins (n = 140; Supplementary Table 3) found by the Benjamini-Hochberg correction between late stage cervical cancer and healthy epithelium were significantly correlated (Wilcoxon rank-sum test, p = 0.002) with ^{2}log transformed expression levels found in the published transcriptomics dataset. Sample names indicate histology of tumor material: NEC - neuroendocrine carcinoma; SCC - squamous cell carcinoma; AC - adenocarcinoma; ASC - adenosquamous carcinoma; CCC - clear cell carcinoma.

Supplementaty Excel Data 1: Concentrations of MCM3, CEACAM5, S100P and ICAM1 in whole tissue lysates

Supplementaty Excel Data 2: Analytical parameters

Supplementary Table 1: List of significant different proteins (*n* = 13) between late stage cervical cancer and healthy epithelium based on Bonferroni correction for multiple testing

Protein name	Gene name	*p*-value	^{2}log fold–change
Keratin, type I cytoskeletal 13	KRT13	1.12E-08	–2.0
Type-1 angiotensin II receptor-associated protein	CRNN	1.75E-07	2.5
Cornulin	DSC2	4.89E-07	–3.1
Flap endonuclease 1	MCM6	2.26E-06	2.5
Desmocollin-2	MCM7	3.41E-06	–1.5
SWI/SNF-related matrix-associated actin-dependent regulator of chromatin subfamily A member 5	ERP29	3.47E-06	2.2
Phosphatidate cytidylyltransferase 2	FEN1	3.71E-05	2.4
DNA replication licensing factor MCM7	CDS2	4.21E-05	3.5
DNA replication licensing factor MCM6	APMAP	4.52E-05	3.0
ADP-dependent glucokinase	ADPGK	5.31E-05	2.8
Adipocyte plasma membrane-associated protein	SMARCA5	9.01E-05	2.3
Endoplasmic reticulum resident protein 29	AGTRAP	1.95E-04	2.2
Oxysterol-binding protein-related protein 3	OSBPL3	3.13E-04	2.9

Zero counts were converted to 0.125 to enable log calculations.

Supplementary Table 2: List of significant different proteins (*n* = 319) between early stage cervical cancer and healthy epithelium using Benjamini-Hochberg correction for multiple testing. See Supplementary_Table_2

Supplementaty Table 3: List of significant different proteins (*n* = 140) between late stage cervical cancer and healthy epithelium using Benjamini-Hochberg correction for multiple testing. See Supplementary_Table_3

Supplementary Table 4: Comparison of mutated or expressed genes found by Ojesina *et al.* with proteins identified by LCM with minimal two peptides (for the healthy epithelium, early and late stage cervical cancer groups) and in cell lines HeLa, U87 and HEK293

Mutated (%)/expressed gene (*)	Identified by LCM methodology	Significant (yes/no) between early stage cervical cancer with healthy epithelium (spectral counting)	Number of unique peptides found in					
			H	EC	LC	HL	U	HK
FBXW7 (15%)	no							
PIK3CA (14%)	no							
MAPK1 (8%)	yes	no	3	3	4	7	6	0
HLA-B (9%)	no							
STK11 (4%)	no							
EP300 (16%)	no							
NFE2L2 (4%)	no							
PTEN (6%)	no							
ELF3 (13%)	no							
CBFB (8%)	yes	no	0	0	0	2	0	0
TP53 (5%)	no							
ERBB2 (6%)	no							
PARN	yes	no	0	2	0	4	0	0
KRAS (8%)	no							
RPS6KB1	no							
MAFA	no							
SNIP1	no							
POC1B	no							
BCL11B	no							
IFNG	no							
JAK2	no							
TP63	yes	no	2	3	3	0	0	0
MYC	no							
FANCC	no							
RAD51B	no							
CEACAM5	yes	yes, $p = 0.007$	0	6	4	0	0	0
TNIK	no							
GLI2	no							
NR4A2	no							
PROX1	no							
EIF2C2	no							
FAM179B	no							
SERPINB4	no							

*Genes expressed in percentages indicate mutation rate.
H: healthy cervical epithelium.
EC: early stage cervical cancer.
LC: late stage cervical cancer.
HL: HeLa.
U: U87.
HK: HEK293.

Supplementary Table 5: Selected transitions for each peptide (endogenous and SIL) corresponding to *MCM3, CEACAM5, S100P, ICAM1* to perform quantitative LC-MS/MS assays by PRM

**MCM3 peptide 1**	**y5**	**y6**	**y7**
LIVNVNDLR (*m/z* 528.31, +2)	616.34	730.38	829.45
LIVNVNDLR (SIL) (*m/z* 533.32, +2)	626.35	740.39	839.46
**MCM3 peptide 2**	**y7**	**y8**	**y9**
SVDVILDDDLVDK (*m/z* 723.37, +2)	819.37	932.46	1045.54
SVDVILDDDLVDK (SIL) (*m/z* 727.38, +2)	827.39	940.47	1053.56
**MCM3 peptide 3**	**y4**	**y5**	**y6**
GGYTSGTFR (*m/z* 473.22, +2)	480.26	567.29	668.34
GGYTSGTFR (SIL) (*m/z* 478.23, +2)	490.26	577.30	678.34
**CEACAM5 peptide 1**	**y7**	**y8**	**y9**
SDLVNEEATGQFR (*m/z* 733.35, +2)	808.40	937.44	1051.48
SDLVNEEATGQFR (SIL) (*m/z* 738.35, +2)	818.40	947.45	1061.49
**CEACAM5 peptide 2**	**y5**	**y6**	**y7**
TLTLLSVTR (*m/z* 502.31, +2)	575.35	688.44	789.48
TLTLLSVTR (SIL) (*m/z* 507.32, +2)	585.36	698.44	799.49
**S100P peptide 1**	**y8**	**y9**	**y11**
YSGSEGSTQTLTK (*m/z* 679.83, +2)	835.45	964.49	1108.55
YSGSEGSTQTLTK (SIL) (*m/z* 683.83, +2)	843.47	972.51	1116.56
**S100P peptide 2**	**y6**	**y7**	**y8**
ELPGFLQSGK (*m/z* 538.29, +2)	679.38	736.40	833.45
ELPGFLQSGK (SIL) (*m/z* 542.30, +2)	687.39	744.41	841.47
**ICAM1 peptide 1**	**y7**	**y8**	**y9**
ASVSVTAEDEGTQR (*m/z* 725.34, +2)	834.36	905.40	1006.44
ASVSVTAEDEGTQR (SIL) (*m/z* 730.35, +2)	844.37	915.40	1016.45
**ICAM1 peptide 2**	**y8**	**y10**	**y12**
DGTFPLPIGESVTVTR (*m/z* 844.95, +2)	848.45	1058.58	1268.72
DGTFPLPIGESVTVTR (SIL) (*m/z* 849.95, +2)	858.46	1068.59	1278.73
