# Supplementary material for: Proteomic alterations in early stage cervical cancer
Source: Oncotarget. 2018 Apr 6;9(26):18128–47. doi: 10.18632/oncotarget.24773 (PMC5915062; doi:10.18632/oncotarget.24773)
Supplement: Supplementary file 4 [file oncotarget-09-18128-s004.docx]

**Table S2.** List of significant different proteins (*n* = 319) between early stage cervical cancer and healthy epithelium using Benjamini-Hochberg correction for multiple testing. Zero counts were converted to 0.125 to enable log calculations.

| Protein name | Gene name | p-value | ^2^log fold-change |
| --- | --- | --- | --- |
| Keratin, type I cytoskeletal 13 | KRT13 | 1.50E-09 | -2.6 |
| Cluster of Envoplakin | EVPL | 1.18E-07 | -1.9 |
| Cornulin | CRNN | 1.37E-07 | -4.6 |
| Desmocollin-2 | DSC2 | 4.47E-07 | -1.7 |
| Cluster of Acidic leucine-rich nuclear phosphoprotein 32 family member A | ANP32A | 1.57E-06 | 1.4 |
| DNA topoisomerase 1 | TOP1 | 6.56E-06 | 2.1 |
| Cluster of Keratin, type II cytoskeletal 73 | KRT73 | 7.64E-06 | -1.0 |
| Protein disulfide-isomerase TMX3 | TMX3 | 1.13E-05 | 2.0 |
| Protein disulfide-isomerase A3 | PDIA3 | 1.42E-05 | 0.7 |
| DNA replication licensing factor MCM4 | MCM4 | 1.92E-05 | 5.1 |
| Small proline-rich protein 3 | SPRR3 | 1.93E-05 | -2.4 |
| DNA replication licensing factor MCM6 | MCM6 | 1.95E-05 | 3.0 |
| DNA replication licensing factor MCM7 | MCM7 | 2.04E-05 | 3.6 |
| Desmoglein-1 | DSG1 | 2.12E-05 | -2.9 |
| Ribosome-binding protein 1 | RRBP1 | 2.15E-05 | 1.4 |
| Exportin-2 | CSE1L | 2.45E-05 | 1.2 |
| DNA replication licensing factor MCM3 | MCM3 | 2.55E-05 | -1.5 |
| EF-hand domain-containing protein D2 | EFHD2 | 2.68E-05 | 4.3 |
| Periplakin | PPL | 3.01E-05 | 1.5 |
| Heterogeneous nuclear ribonucleoproteins A2/B1 | HNRNPA2B1 | 3.31E-05 | -1.4 |
| Cluster of Keratin, type II cytoskeletal 5 | KRT5 | 3.31E-05 | 1.0 |
| 78 kDa glucose-regulated protein | HSPA5 | 3.60E-05 | 0.8 |
| Involucrin | IVL | 3.86E-05 | -2.0 |
| Calreticulin | CALR | 4.23E-05 | 1.5 |
| Nucleoprotein TPR | TPR | 4.28E-05 | 1.8 |
| DNA replication licensing factor MCM2 | MCM2 | 5.24E-05 | 4.9 |
| Junction plakoglobin | JUP | 5.58E-05 | -0.9 |
| Thioredoxin-related transmembrane protein 1 | TMX1 | 5.86E-05 | 1.7 |
| Nuclear pore complex protein Nup93 | NUP93 | 6.13E-05 | 1.7 |
| Keratin, type II cytoskeletal 78 | KRT78 | 6.16E-05 | -1.8 |
| Filamin-A | FLNA | 6.55E-05 | 0.8 |
| Heat shock protein 105 kDa | HSPH1 | 6.59E-05 | 1.3 |
| Four and a half LIM domains protein 2 | FHL2 | 7.80E-05 | 2.1 |
| Endoplasmic reticulum resident protein 29 | ERP29 | 7.93E-05 | 1.8 |
| Keratin, type II cytoskeletal 4 | KRT4 | 8.00E-05 | -2.0 |
| Nuclear pore complex protein Nup155 | NUP155 | 9.16E-05 | 1.9 |
| Talin-1 | TLN1 | 9.23E-05 | 1.4 |
| ATP-dependent RNA helicase A | DHX9 | 9.69E-05 | 0.8 |
| Chloride intracellular channel protein 3 | CLIC3 | 1.12E-04 | -1.8 |
| Protein disulfide-isomerase A4 | PDIA4 | 1.13E-04 | 1.4 |
| Phospholipase A-2-activating protein | PLAA | 1.15E-04 | 3.2 |
| Eukaryotic translation initiation factor 2 subunit 3 | EIF2S3 | 1.15E-04 | 1.5 |
| Alpha-2-macroglobulin-like protein 1 | A2ML1 | 1.17E-04 | -2.2 |
| Suprabasin | SBSN | 1.22E-04 | -2.7 |
| Keratin, type II cytoskeletal 1 | KRT1 | 1.36E-04 | -1.2 |
| Keratin, type II cytoskeletal 72 | KRT72 | 1.37E-04 | -0.9 |
| Protein disulfide-isomerase A6 | PDIA6 | 1.38E-04 | 0.9 |
| Keratin, type I cytoskeletal 15 | KRT15 | 1.44E-04 | -1.4 |
| Flap endonuclease 1 | FEN1 | 1.49E-04 | 2.7 |
| DNA-dependent protein kinase catalytic subunit | PRKDC | 1.70E-04 | 1.2 |
| Keratin, type II cytoskeletal 1b | KRT77 | 1.74E-04 | -1.2 |
| Tryptophan--tRNA ligase, cytoplasmic | WARS | 1.90E-04 | 3.0 |
| Eukaryotic translation initiation factor 4 gamma 2 | EIF4G2 | 2.05E-04 | 1.9 |
| DnaJ homolog subfamily C member 7 | DNAJC7 | 2.12E-04 | 1.3 |
| Reticulocalbin-1 | RCN1 | 2.23E-04 | 2.2 |
| FACT complex subunit SPT16 | SUPT16H | 2.28E-04 | 1.8 |
| Serine/arginine-rich splicing factor 1 | SRSF1 | 2.30E-04 | 1.8 |
| tRNA (cytosine(34)-C(5))-methyltransferase | NSUN2 | 2.43E-04 | 2.0 |
| Cluster of Endoplasmin | HSP90B1 | 2.47E-04 | 0.7 |
| Antigen peptide transporter 1 | TAP1 | 2.60E-04 | 2.5 |
| Desmoplakin | DSP | 2.60E-04 | -0.7 |
| Solute carrier family 2, facilitated glucose transporter member 1 | SLC2A1 | 2.70E-04 | 2.4 |
| Double-stranded RNA-specific adenosine deaminase | ADAR | 2.88E-04 | 2.7 |
| Ubiquitin-like modifier-activating enzyme 1 | UBA1 | 2.91E-04 | 1.0 |
| Cluster of Alpha-actinin-1 | ACTN1 | 2.99E-04 | 3.2 |
| Pre-mRNA-processing factor 6 | PRPF6 | 3.06E-04 | 2.4 |
| Replication protein A 70 kDa DNA-binding subunit | RPA1 | 3.09E-04 | 1.2 |
| Fascin | FSCN1 | 3.12E-04 | 1.1 |
| Protein S100-P | S100P | 3.13E-04 | 2.9 |
| DNA polymerase delta catalytic subunit | POLD1 | 3.13E-04 | 2.9 |
| Serpin H1 | SERPINH1 | 3.30E-04 | 3.7 |
| Structural maintenance of chromosomes protein 3 | SMC3 | 3.35E-04 | 1.4 |
| Eukaryotic initiation factor 4A-III | EIF4A3 | 3.52E-04 | 0.8 |
| DNA (cytosine-5)-methyltransferase 1 | DNMT1 | 3.58E-04 | 2.5 |
| Tripartite motif-containing protein 29 | TRIM29 | 3.58E-04 | -0.9 |
| Dolichyl-diphosphooligosaccharide--protein glycosyltransferase subunit 1 | RPN1 | 3.80E-04 | 0.8 |
| Cluster of Tubulin beta chain | TUBB | 4.01E-04 | 3.2 |
| Procollagen galactosyltransferase 1 | COLGALT1 | 4.13E-04 | 2.5 |
| Poly(U)-binding-splicing factor PUF60 | PUF60 | 4.19E-04 | 1.8 |
| Putative pre-mRNA-splicing factor ATP-dependent RNA helicase DHX15 | DHX15 | 4.38E-04 | 2.3 |
| Proliferating cell nuclear antigen | PCNA | 4.38E-04 | -1.1 |
| Plakophilin-1 | PKP1 | 4.40E-04 | 2.5 |
| DnaJ homolog subfamily C member 9 | DNAJC9 | 4.40E-04 | 2.7 |
| Septin-11 | SEPT11 | 4.57E-04 | 1.8 |
| Thymidine phosphorylase | TYMP | 4.66E-04 | 2.2 |
| Protein DEK | DEK | 4.74E-04 | 1.7 |
| BolA-like protein 2 | BOLA2 | 4.91E-04 | 1.0 |
| Gamma-interferon-inducible protein 16 | IFI16 | 5.00E-04 | 1.8 |
| Splicing factor 3A subunit 1 | SF3A1 | 5.09E-04 | 2.3 |
| Acyl-coenzyme A thioesterase 13 | ACOT13 | 5.09E-04 | 2.3 |
| Phosphatidate cytidylyltransferase 2 | CDS2 | 5.32E-04 | 0.9 |
| Cluster of Rho-related GTP-binding protein RhoG | RHOG | 5.39E-04 | 2.8 |
| Vesicle-trafficking protein SEC22b | SEC22B | 5.55E-04 | 0.9 |
| Protein RCC2 | RCC2 | 5.61E-04 | 1.5 |
| 26S proteasome non-ATPase regulatory subunit 2 | PSMD2 | 5.79E-04 | 3.2 |
| C-1-tetrahydrofolate synthase, cytoplasmic | MTHFD1 | 5.79E-04 | 1.8 |
| BTB/POZ domain-containing protein KCTD12 | KCTD12 | 6.01E-04 | 2.5 |
| Putative RNA-binding protein Luc7-like 2 | LUC7L2 | 6.10E-04 | 1.9 |
| Structural maintenance of chromosomes protein 1A | SMC1A | 6.19E-04 | 1.0 |
| H/ACA ribonucleoprotein complex subunit 4 | DKC1 | 6.22E-04 | 1.9 |
| Eukaryotic translation initiation factor 2 subunit 1 | EIF2S1 | 6.26E-04 | 0.8 |
| Serrate RNA effector molecule homolog | SRRT | 6.26E-04 | 1.8 |
| Reticulocalbin-2 | RCN2 | 6.30E-04 | 1.4 |
| Splicing factor 3B subunit 3 | SF3B3 | 6.33E-04 | 2.6 |
| HIV Tat-specific factor 1 | HTATSF1 | 6.43E-04 | 2.4 |
| Alanine--tRNA ligase, cytoplasmic | AARS | 6.46E-04 | 1.1 |
| Probable ATP-dependent RNA helicase DDX5 | DDX5 | 6.55E-04 | -1.3 |
| Spectrin beta chain, non-erythrocytic 1 | SPTBN1 | 6.57E-04 | 1.1 |
| Tenascin | TNC | 7.01E-04 | 4.3 |
| Cluster of Clathrin heavy chain 1 | CLTC | 7.01E-04 | 0.6 |
| Cluster of Protein transport protein Sec61 subunit alpha isoform 1 | SEC61A1 | 7.11E-04 | 1.5 |
| Acidic leucine-rich nuclear phosphoprotein 32 family member B | ANP32B | 7.33E-04 | 1.2 |
| Hypoxia up-regulated protein 1 | HYOU1 | 7.35E-04 | 1.5 |
| Splicing factor 3A subunit 3 | SF3A3 | 7.47E-04 | 1.5 |
| Nicotinamide phosphoribosyltransferase | NAMPT | 7.49E-04 | 2.1 |
| Regulation of nuclear pre-mRNA domain-containing protein 1B | RPRD1B | 7.70E-04 | 1.3 |
| ERO1-like protein alpha | ERO1L | 8.29E-04 | 0.6 |
| DNA replication licensing factor MCM5 | MCM5 | 8.37E-04 | 2.4 |
| V-type proton ATPase subunit d 1 | ATP6V0D1 | 8.60E-04 | 3.0 |
| Heterogeneous nuclear ribonucleoprotein K | HNRNPK | 8.65E-04 | 2.5 |
| Tubulin-folding cofactor B | TBCB | 8.70E-04 | 0.7 |
| DNA topoisomerase 2-alpha | TOP2A | 9.88E-04 | 2.1 |
| Staphylococcal nuclease domain-containing protein 1 | SND1 | 1.03E-03 | 0.8 |
| Poly [ADP-ribose] polymerase 1 | PARP1 | 1.09E-03 | 3.1 |
| Rho GTPase-activating protein 1 | ARHGAP1 | 1.10E-03 | 1.0 |
| Complement C1q subcomponent subunit C | C1QC | 1.11E-03 | 1.3 |
| Rab GDP dissociation inhibitor alpha | GDI1 | 1.13E-03 | 1.0 |
| Transcription intermediary factor 1-beta | TRIM28 | 1.14E-03 | 2.8 |
| HLA class II histocompatibility antigen, DR alpha chain | HLA-DRA | 1.17E-03 | 1.0 |
| Ubiquitin carboxyl-terminal hydrolase 14 | USP14 | 1.21E-03 | 0.9 |
| DnaJ homolog subfamily C member 13 | DNAJC13 | 1.21E-03 | 2.4 |
| Replication factor C subunit 2 | RFC2 | 1.21E-03 | 1.1 |
| Serine protease inhibitor Kazal-type 5 | SPINK5 | 1.21E-03 | 3.4 |
| NSFL1 cofactor p47 | NSFL1C | 1.21E-03 | 3.4 |
| Apolipoprotein L2 | APOL2 | 1.26E-03 | -4.0 |
| Mitochondrial import inner membrane translocase subunit TIM50 | TIMM50 | 1.35E-03 | 0.9 |
| Cluster of Interferon-induced guanylate-binding protein 1 | GBP1 | 1.37E-03 | 2.6 |
| Transforming acidic coiled-coil-containing protein 2 | TACC2 | 1.37E-03 | 1.8 |
| Replication protein A 32 kDa subunit | RPA2 | 1.38E-03 | 3.6 |
| Inactive tyrosine-protein kinase 7 | PTK7 | 1.40E-03 | -1.8 |
| Synembryn-A | RIC8A | 1.42E-03 | 2.8 |
| Cluster of Protein FAM49B | FAM49B | 1.42E-03 | 2.8 |
| UDP-glucose:glycoprotein glucosyltransferase 1 | UGGT1 | 1.42E-03 | 2.8 |
| Histone acetyltransferase type B catalytic subunit | HAT1 | 1.43E-03 | 1.3 |
| Cluster of Keratin, type I cytoskeletal 10 | KRT10 | 1.47E-03 | 2.4 |
| Coiled-coil-helix-coiled-coil-helix domain-containing protein 3, mitochondrial | CHCHD3 | 1.52E-03 | 2.7 |
| ER membrane protein complex subunit 1 | EMC1 | 1.55E-03 | -0.9 |
| Proteasome subunit beta type-9 | PSMB9 | 1.56E-03 | 2.1 |
| Calcium-binding mitochondrial carrier protein Aralar2 | SLC25A13 | 1.56E-03 | 2.8 |
| Regulator of chromosome condensation | RCC1 | 1.62E-03 | 2.1 |
| Lamin-B1 | LMNB1 | 1.62E-03 | 1.8 |
| Cluster of Cornifin-B | SPRR1B | 1.63E-03 | 2.6 |
| Gelsolin | GSN | 1.64E-03 | 0.6 |
| Adipocyte plasma membrane-associated protein | APMAP | 1.70E-03 | 4.0 |
| Cluster of Ig heavy chain V-II region ARH-77 | Ig heavy chain V-II region ARH-77 | 1.71E-03 | 0.7 |
| Eukaryotic translation initiation factor 4 gamma 1 | EIF4G1 | 1.72E-03 | 2.3 |
| Cluster of V-type proton ATPase subunit B, brain isoform | ATP6V1B2 | 1.75E-03 | 2.0 |
| NEDD8-conjugating enzyme Ubc12 | UBE2M | 1.75E-03 | 1.0 |
| YTH domain family protein 2 | YTHDF2 | 1.75E-03 | 1.0 |
| Protein transport protein Sec31A | SEC31A | 1.75E-03 | 1.1 |
| Transformer-2 protein homolog beta | TRA2B | 1.75E-03 | 1.1 |
| Cytospin-B | SPECC1 | 1.77E-03 | 1.6 |
| U6 snRNA-associated Sm-like protein LSm2 | LSM2 | 1.85E-03 | 1.2 |
| ADP-dependent glucokinase | ADPGK | 1.88E-03 | 1.2 |
| Cellular retinoic acid-binding protein 2 | CRABP2 | 1.88E-03 | 2.4 |
| Cluster of Heterogeneous nuclear ribonucleoproteins C1/C2 | HNRNPC | 1.88E-03 | 2.4 |
| Guanylate-binding protein 6 | GBP6 | 1.88E-03 | 2.4 |
| Eyes absent homolog 3 | EYA3 | 1.89E-03 | -1.2 |
| Polypeptide N-acetylgalactosaminyltransferase 2 | GALNT2 | 1.89E-03 | 0.7 |
| PEST proteolytic signal-containing nuclear protein | PCNP | 1.95E-03 | -2.5 |
| Tyrosine--tRNA ligase, cytoplasmic | YARS | 1.96E-03 | 2.9 |
| Cluster of HLA class II histocompatibility antigen, DRB1-16 beta chain | HLA-DRB1 | 1.99E-03 | 3.0 |
| Heterogeneous nuclear ribonucleoprotein L | HNRNPL | 1.99E-03 | 3.0 |
| Desmoglein-3 | DSG3 | 1.99E-03 | 1.6 |
| Importin subunit beta-1 | KPNB1 | 2.08E-03 | 0.4 |
| Elongation factor Tu, mitochondrial | TUFM | 2.12E-03 | 0.7 |
| Moesin | MSN | 2.19E-03 | -0.7 |
| Filamin-C | FLNC | 2.21E-03 | 1.3 |
| Protein PML | PML | 2.21E-03 | 1.1 |
| Cadherin-13 | CDH13 | 2.21E-03 | 0.8 |
| Vinculin | VCL | 2.22E-03 | 0.8 |
| Alpha-parvin | PARVA | 2.31E-03 | 1.9 |
| Signal transducer and activator of transcription 1-alpha/beta | STAT1 | 2.32E-03 | -1.3 |
| Glucosidase 2 subunit beta | PRKCSH | 2.34E-03 | 0.6 |
| Annexin A3 | ANXA3 | 2.39E-03 | 1.6 |
| Collagen alpha-1(VII) chain | COL7A1 | 2.41E-03 | 2.5 |
| Septin-9 | SEPT9 | 2.41E-03 | 0.9 |
| Delta(24)-sterol reductase | DHCR24 | 2.41E-03 | 2.1 |
| Stathmin | STMN1 | 2.42E-03 | -2.7 |
| Splicing factor 3B subunit 4 | SF3B4 | 2.45E-03 | 1.8 |
| SUMO-activating enzyme subunit 2 | UBA2 | 2.49E-03 | 2.6 |
| Dynamin-1-like protein | DNM1L | 2.51E-03 | 3.3 |
| Cluster of Serine/threonine-protein kinase PAK 2 | PAK2 | 2.55E-03 | 1.7 |
| Integrin beta-1 | ITGB1 | 2.56E-03 | 2.1 |
| Deoxynucleoside triphosphate triphosphohydrolase SAMHD1 | SAMHD1 | 2.62E-03 | 1.6 |
| Sialic acid synthase | NANS | 2.65E-03 | 3.3 |
| RNA-binding protein 25 | RBM25 | 2.74E-03 | 1.4 |
| Poly(U)-specific endoribonuclease | ENDOU | 2.74E-03 | 2.1 |
| RNA-binding protein with serine-rich domain 1 | RNPS1 | 2.75E-03 | 2.1 |
| NADH dehydrogenase [ubiquinone] iron-sulfur protein 2, mitochondrial | NDUFS2 | 2.80E-03 | -3.4 |
| Monocarboxylate transporter 2 | SLC16A7 | 2.81E-03 | -2.3 |
| Keratinocyte differentiation-associated protein | KRTDAP | 2.81E-03 | -2.3 |
| Brain-specific angiogenesis inhibitor 1-associated protein 2 | BAIAP2 | 2.81E-03 | -2.3 |
| GMP synthase [glutamine-hydrolyzing] | GMPS | 2.81E-03 | 0.9 |
| Protein phosphatase 1G | PPM1G | 2.82E-03 | -2.1 |
| Nuclear autoantigenic sperm protein | NASP | 2.88E-03 | 3.8 |
| E3 ubiquitin-protein ligase DTX3L | DTX3L | 2.94E-03 | 1.8 |
| Importin subunit alpha-2 | KPNA2 | 3.00E-03 | 3.0 |
| Superkiller viralicidic activity 2-like 2 | SKIV2L2 | 3.00E-03 | 3.7 |
| IST1 homolog | IST1 | 3.08E-03 | 3.8 |
| Bifunctional coenzyme A synthase | COASY | 3.09E-03 | 1.6 |
| E3 ubiquitin-protein ligase HUWE1 | HUWE1 | 3.25E-03 | 2.4 |
| Calumenin | CALU | 3.26E-03 | 1.8 |
| Aspartyl/asparaginyl beta-hydroxylase | ASPH | 3.26E-03 | 1.4 |
| Glutamine--fructose-6-phosphate aminotransferase [isomerizing] 1 | GFPT1 | 3.28E-03 | 3.5 |
| Malectin | MLEC | 3.30E-03 | 1.3 |
| CDGSH iron-sulfur domain-containing protein 2 | CISD2 | 3.40E-03 | 1.6 |
| Cluster of ATP-dependent RNA helicase DDX3X | DDX3X | 3.43E-03 | 1.7 |
| Serine/arginine repetitive matrix protein 2 | SRRM2 | 3.43E-03 | 2.3 |
| Cytosol aminopeptidase | LAP3 | 3.50E-03 | 0.8 |
| Cluster of Sarcoplasmic/endoplasmic reticulum calcium ATPase 2 | ATP2A2 | 3.52E-03 | 2.0 |
| Medium-chain specific acyl-CoA dehydrogenase, mitochondrial | ACADM | 3.61E-03 | 2.6 |
| Proteasome subunit alpha type-4 | PSMA4 | 3.67E-03 | 1.0 |
| ATP-dependent RNA helicase DDX1 | DDX1 | 3.81E-03 | 1.6 |
| ER membrane protein complex subunit 3 | EMC3 | 3.83E-03 | 2.6 |
| DnaJ homolog subfamily A member 1 | DNAJA1 | 3.85E-03 | 1.0 |
| Epiplakin | EPPK1 | 3.96E-03 | 1.6 |
| Flavin reductase (NADPH) | BLVRB | 3.98E-03 | 1.6 |
| Replication factor C subunit 3 | RFC3 | 4.01E-03 | 2.0 |
| Desmoglein-2 | DSG2 | 4.02E-03 | 1.7 |
| Septin-7 | SEPT7 | 4.04E-03 | 2.2 |
| Transferrin receptor protein 1 | TFRC | 4.12E-03 | 1.2 |
| Apoptosis-inducing factor 1, mitochondrial | AIFM1 | 4.13E-03 | 1.1 |
| SWI/SNF complex subunit SMARCC1 | SMARCC1 | 4.21E-03 | 2.6 |
| Cluster of Histone-binding protein RBBP4 | RBBP4 | 4.28E-03 | 1.3 |
| Unconventional myosin-VI | MYO6 | 4.38E-03 | 1.8 |
| Pre-mRNA-processing factor 19 | PRPF19 | 4.43E-03 | 1.8 |
| CTP synthase 1 | CTPS1 | 4.45E-03 | 2.8 |
| Prolyl 3-hydroxylase 1 | LEPRE1 | 4.49E-03 | 1.5 |
| Peptidyl-tRNA hydrolase 2, mitochondrial | PTRH2 | 4.58E-03 | 2.6 |
| 15 kDa selenoprotein | SEP15 | 4.58E-03 | 2.6 |
| Intercellular adhesion molecule 1 | ICAM1 | 4.58E-03 | 2.6 |
| Coatomer subunit beta' | COPB2 | 4.58E-03 | 2.6 |
| Sorting nexin-6 | SNX6 | 4.75E-03 | 3.6 |
| Endoplasmic reticulum aminopeptidase 1 | ERAP1 | 4.81E-03 | 1.1 |
| Translocon-associated protein subunit gamma | SSR3 | 4.88E-03 | 1.8 |
| Golgi phosphoprotein 3 | GOLPH3 | 5.03E-03 | 2.4 |
| DNA replication complex GINS protein PSF3 | GINS3 | 5.04E-03 | 1.5 |
| Cyclin-dependent kinase inhibitor 1 | CDKN1A | 5.06E-03 | 1.8 |
| Cluster of Histone H3.3 | H3F3A | 5.06E-03 | 1.8 |
| Aldehyde dehydrogenase family 16 member A1 | ALDH16A1 | 5.06E-03 | 1.8 |
| EH domain-containing protein 4 | EHD4 | 5.06E-03 | 1.8 |
| Nuclear pore complex protein Nup153 | NUP153 | 5.06E-03 | 1.8 |
| Alpha-enolase | ENO1 | 5.07E-03 | 1.8 |
| Cluster of Tropomyosin alpha-4 chain | TPM4 | 5.12E-03 | 2.0 |
| FACT complex subunit SSRP1 | SSRP1 | 5.13E-03 | 2.9 |
| Threonine--tRNA ligase, cytoplasmic | TARS | 5.15E-03 | 0.6 |
| Heterogeneous nuclear ribonucleoprotein A3 | HNRNPA3 | 5.18E-03 | 2.2 |
| RNA-binding protein 10 | RBM10 | 5.25E-03 | 1.8 |
| Acyl-coenzyme A thioesterase 9, mitochondrial | ACOT9 | 5.39E-03 | 0.7 |
| Cdc42 effector protein 4 | CDC42EP4 | 5.41E-03 | 3.0 |
| Poly [ADP-ribose] polymerase 9 | PARP9 | 5.42E-03 | 3.0 |
| Protein canopy homolog 2 | CNPY2 | 5.47E-03 | 1.8 |
| Lactotransferrin | LTF | 5.66E-03 | 3.7 |
| Superoxide dismutase [Mn], mitochondrial | SOD2 | 5.72E-03 | 1.7 |
| Sodium/potassium-transporting ATPase subunit beta-1 | ATP1B1 | 5.80E-03 | 5.1 |
| Epoxide hydrolase 1 | EPHX1 | 5.91E-03 | 2.2 |
| Septin-6 | SEPT6 | 5.92E-03 | 2.8 |
| Growth factor receptor-bound protein 2 | GRB2 | 6.02E-03 | 1.4 |
| Protein SAAL1 | SAAL1 | 6.06E-03 | 3.0 |
| Cytosolic 5'-nucleotidase 3A | NT5C3A | 6.08E-03 | 2.3 |
| Phospholipid scramblase 1 | PLSCR1 | 6.08E-03 | 2.3 |
| Nicotinamide N-methyltransferase | NNMT | 6.08E-03 | 2.3 |
| 28S ribosomal protein S18b, mitochondrial | MRPS18B | 6.08E-03 | 2.3 |
| Nuclear pore membrane glycoprotein 210 | NUP210 | 6.08E-03 | 2.3 |
| Phosphoglycerate kinase 1 | PGK1 | 6.08E-03 | 2.3 |
| Cluster of Metastasis-associated protein MTA2 | MTA2 | 6.09E-03 | 4.0 |
| Endoplasmic reticulum metallopeptidase 1 | ERMP1 | 6.20E-03 | 4.0 |
| Putative RNA-binding protein Luc7-like 1 | LUC7L | 6.27E-03 | 0.8 |
| Estradiol 17-beta-dehydrogenase 12 | HSD17B12 | 6.34E-03 | 2.1 |
| E3 ubiquitin-protein ligase RNF213 | RNF213 | 6.37E-03 | 2.1 |
| Unconventional myosin-Ib | MYO1B | 6.38E-03 | 1.6 |
| Asparagine--tRNA ligase, cytoplasmic | NARS | 6.40E-03 | 2.2 |
| Cluster of Ribose-phosphate pyrophosphokinase 2 | PRPS2 | 6.50E-03 | 1.7 |
| Proteasome activator complex subunit 1 | PSME1 | 6.60E-03 | 3.0 |
| Cluster of Carcinoembryonic antigen-related cell adhesion molecule 5 | CEACAM5 | 6.82E-03 | 3.5 |
| NAD(P) transhydrogenase, mitochondrial | NNT | 6.85E-03 | 1.5 |
| RNA-binding protein 39 | RBM39 | 7.23E-03 | 1.8 |
| Prostaglandin F2 receptor negative regulator | PTGFRN | 7.26E-03 | 1.8 |
| L-lactate dehydrogenase A chain | LDHA | 7.39E-03 | 2.5 |
| Cluster of SWI/SNF-related matrix-associated actin-dependent regulator of chromatin subfamily A member 5 | SMARCA5 | 7.58E-03 | 2.0 |
| Sister chromatid cohesion protein PDS5 homolog A | PDS5A | 7.67E-03 | 2.4 |
| Importin subunit alpha-1 | KPNA1 | 8.11E-03 | 0.6 |
| Probable ATP-dependent RNA helicase DDX23 | DDX23 | 8.15E-03 | 2.0 |
| Erythrocyte band 7 integral membrane protein | STOM | 8.17E-03 | 2.4 |
| Ras-related protein Rab-7a | RAB7A | 8.25E-03 | 2.8 |
| DNA-directed RNA polymerase II subunit RPB1 | POLR2A | 8.66E-03 | 2.4 |
| Serine/arginine-rich splicing factor 11 | SRSF11 | 8.74E-03 | 1.5 |
| Amyloid beta A4 protein | APP | 8.81E-03 | 1.2 |
| Cluster of Receptor-type tyrosine-protein phosphatase F | PTPRF | 1.02E-02 | 2.2 |
| Dihydropyrimidinase-related protein 3 | DPYSL3 | 1.02E-02 | 2.2 |
| Hexokinase-2 | HK2 | 1.02E-02 | 2.2 |
| Structural maintenance of chromosomes flexible hinge domain-containing protein 1 | SMCHD1 | 1.13E-02 | 3.3 |
| DBIRD complex subunit ZNF326 | ZNF326 | 1.18E-02 | 4.0 |
| Nuclear pore complex protein Nup98-Nup96 | NUP98 | 1.19E-02 | 3.3 |
| DNA ligase 1 | LIG1 | 1.19E-02 | 2.9 |
| Cluster of AP-2 complex subunit alpha-1 | AP2A1 | 1.19E-02 | 2.9 |
| E3 ubiquitin-protein ligase UBR4 | UBR4 | 1.20E-02 | 2.5 |
| Integrin alpha-V | ITGAV | 1.20E-02 | 2.5 |
| Kanadaptin | SLC4A1AP | 1.23E-02 | 2.8 |
| Extended synaptotagmin-2 | ESYT2 | 1.23E-02 | 2.8 |
| Nuclear pore complex protein Nup88 | NUP88 | 1.23E-02 | 2.8 |
| Monocarboxylate transporter 4 | SLC16A3 | 1.34E-02 | 2.6 |
| Cluster of Heat shock protein HSP 90-alpha | HSP90AA1 | 1.34E-02 | 2.6 |
| WD repeat and HMG-box DNA-binding protein 1 | WDHD1 | 1.34E-02 | 2.6 |
| E3 ubiquitin-protein ligase BRE1A | RNF20 | 1.36E-02 | 2.7 |
| Mitochondrial Rho GTPase 2 | RHOT2 | 1.43E-02 | 2.8 |
| Sister chromatid cohesion protein PDS5 homolog B | PDS5B | 1.45E-02 | 2.8 |
| Cluster of Tropomyosin alpha-3 chain | TPM3 | 2.54E-02 | 0.5 |
