# Supplementary material for: Proteomic alterations in early stage cervical cancer
Source: Oncotarget. 2018 Apr 6;9(26):18128–47. doi: 10.18632/oncotarget.24773 (PMC5915062; doi:10.18632/oncotarget.24773)
Supplement: Supplementary file 5 [file oncotarget-09-18128-s005.docx]

**Table S3.** List of significant different proteins (*n* = 140) between late stage cervical cancer and healthy epithelium using Benjamini-Hochberg correction for multiple testing. Zero counts were converted to 0.125 to enable log calculations.

| Protein name | Gene name | p-value | ^2^log fold-change |
| --- | --- | --- | --- |
| Keratin, type I cytoskeletal 13 | KRT13 | 1.12E-08 | -2.0 |
| Type-1 angiotensin II receptor-associated protein | AGTRAP | 1.75E-07 | 2.5 |
| Cornulin | CRNN | 4.89E-07 | -3.1 |
| Flap endonuclease 1 | FEN1 | 2.26E-06 | 2.5 |
| Desmocollin-2 | DSC2 | 3.41E-06 | -1.5 |
| Cluster of SWI/SNF-related matrix-associated actin-dependent regulator of chromatin subfamily A member 5 | SMARCA5 | 3.47E-06 | 2.2 |
| Keratin, type II cytoskeletal 1b | KRT77 | 1.12E-05 | -1.3 |
| Small proline-rich protein 3 | SPRR3 | 2.06E-05 | -2.3 |
| Alkyldihydroxyacetonephosphate synthase, peroxisomal | AGPS | 3.35E-05 | 2.0 |
| Phosphatidate cytidylyltransferase 2 | CDS2 | 3.71E-05 | 2.4 |
| DNA replication licensing factor MCM7 | MCM7 | 4.21E-05 | 3.5 |
| DNA replication licensing factor MCM6 | MCM6 | 4.52E-05 | 3.0 |
| Junction plakoglobin | JUP | 4.52E-05 | -0.8 |
| ADP-dependent glucokinase | ADPGK | 5.31E-05 | 2.8 |
| Keratin, type II cytoskeletal 1 | KRT1 | 7.37E-05 | -0.9 |
| Adipocyte plasma membrane-associated protein | APMAP | 9.01E-05 | 2.3 |
| Nuclear pore complex protein Nup155 | NUP155 | 1.06E-04 | 1.7 |
| Suprabasin | SBSN | 1.06E-04 | -2.8 |
| Apolipoprotein A-II | APOA2 | 1.47E-04 | -2.3 |
| Cellular retinoic acid-binding protein 2 | CRABP2 | 1.50E-04 | -1.7 |
| Transcription intermediary factor 1-beta | TRIM28 | 1.56E-04 | 1.0 |
| Chloride intracellular channel protein 3 | CLIC3 | 1.65E-04 | -1.7 |
| Proliferating cell nuclear antigen | PCNA | 1.82E-04 | 2.2 |
| DNA-dependent protein kinase catalytic subunit | PRKDC | 1.87E-04 | 1.1 |
| Endoplasmic reticulum resident protein 29 | ERP29 | 1.95E-04 | 2.2 |
| Serine/threonine-protein phosphatase PGAM5, mitochondrial | PGAM5 | 2.05E-04 | 2.3 |
| Ribosome-binding protein 1 | RRBP1 | 2.07E-04 | 1.0 |
| RNA methyltransferase-like protein 1 | RNMTL1 | 2.08E-04 | -2.0 |
| Serine hydroxymethyltransferase, mitochondrial | SHMT2 | 2.11E-04 | 1.9 |
| Replication factor C subunit 3 | RFC3 | 2.13E-04 | 2.4 |
| Chloride intracellular channel protein 4 | CLIC4 | 2.17E-04 | 1.2 |
| Protein S100-A16 | S100A16 | 2.22E-04 | -1.3 |
| Cluster of Acidic leucine-rich nuclear phosphoprotein 32 family member A | ANP32A | 2.68E-04 | 1.1 |
| C-1-tetrahydrofolate synthase, cytoplasmic | MTHFD1 | 2.85E-04 | 1.2 |
| Oxysterol-binding protein-related protein 3 | OSBPL3 | 3.13E-04 | 2.9 |
| Cluster of Envoplakin | EVPL | 3.48E-04 | -1.3 |
| 2'-5'-oligoadenylate synthase 3 | OAS3 | 3.60E-04 | 2.8 |
| Poly(U)-binding-splicing factor PUF60 | PUF60 | 3.72E-04 | 2.2 |
| Heterogeneous nuclear ribonucleoproteins A2/B1 | HNRNPA2B1 | 3.94E-04 | 0.8 |
| Protein disulfide-isomerase A3 | PDIA3 | 4.15E-04 | 0.6 |
| Transforming acidic coiled-coil-containing protein 2 | TACC2 | 4.17E-04 | -2.2 |
| Protein S100-P | S100P | 4.23E-04 | 2.6 |
| Leucine-rich repeat-containing protein 16A | LRRC16A | 4.23E-04 | 2.6 |
| CAD protein | CAD | 4.37E-04 | 2.3 |
| DNA replication licensing factor MCM2 | MCM2 | 4.57E-04 | 5.0 |
| Involucrin | IVL | 4.63E-04 | -1.6 |
| Desmoglein-1 | DSG1 | 4.77E-04 | -1.7 |
| Cluster of Importin subunit alpha-7 | KPNA6 | 4.96E-04 | 1.9 |
| Serine/arginine-rich splicing factor 1 | SRSF1 | 5.04E-04 | 1.8 |
| Ras-related protein Rab-7a | RAB7A | 5.27E-04 | 1.4 |
| Antigen KI-67 | MKI67 | 5.38E-04 | 1.8 |
| Putative RNA-binding protein Luc7-like 1 | LUC7L | 5.49E-04 | 2.2 |
| Acidic leucine-rich nuclear phosphoprotein 32 family member E | ANP32E | 5.68E-04 | 1.2 |
| Elongation factor Tu, mitochondrial | TUFM | 6.11E-04 | 1.0 |
| Programmed cell death protein 4 | PDCD4 | 6.15E-04 | -2.1 |
| Putative RNA-binding protein Luc7-like 2 | LUC7L2 | 6.48E-04 | 1.4 |
| Cluster of Tubulin beta chain | TUBB | 6.98E-04 | 0.7 |
| Plastin-1 | PLS1 | 7.24E-04 | 1.7 |
| Dolichyl-diphosphooligosaccharide--protein glycosyltransferase subunit 1 | RPN1 | 7.40E-04 | 0.8 |
| RNA-binding protein 14 | RBM14 | 7.57E-04 | 1.2 |
| EF-hand domain-containing protein D2 | EFHD2 | 8.03E-04 | 1.3 |
| Ubiquitin carboxyl-terminal hydrolase 14 | USP14 | 8.25E-04 | 1.1 |
| Protein ERGIC-53 | LMAN1 | 8.65E-04 | 2.5 |
| Alanine--tRNA ligase, cytoplasmic | AARS | 9.00E-04 | 2.3 |
| Constitutive coactivator of PPAR-gamma-like protein 1 | FAM120A | 9.11E-04 | 1.0 |
| D-3-phosphoglycerate dehydrogenase | PHGDH | 9.18E-04 | -1.1 |
| ERO1-like protein alpha | ERO1L | 9.20E-04 | 2.4 |
| Protein SET | SET | 9.34E-04 | 0.5 |
| Serrate RNA effector molecule homolog | SRRT | 9.44E-04 | 1.7 |
| Protein disulfide-isomerase A4 | PDIA4 | 9.62E-04 | 1.2 |
| Protein RCC2 | RCC2 | 1.04E-03 | 2.7 |
| Serine protease inhibitor Kazal-type 5 | SPINK5 | 1.05E-03 | -4.7 |
| Cluster of Sarcoplasmic/endoplasmic reticulum calcium ATPase 2 | ATP2A2 | 1.10E-03 | 0.8 |
| DNA replication licensing factor MCM3 | MCM3 | 1.10E-03 | 4.3 |
| Serine/arginine-rich splicing factor 7 | SRSF7 | 1.13E-03 | 1.2 |
| DNA topoisomerase 1 | TOP1 | 1.14E-03 | 1.6 |
| Acidic leucine-rich nuclear phosphoprotein 32 family member B | ANP32B | 1.21E-03 | 1.0 |
| Importin subunit alpha-1 | KPNA1 | 1.29E-03 | 2.9 |
| Spectrin beta chain, non-erythrocytic 1 | SPTBN1 | 1.29E-03 | 1.2 |
| DNA replication licensing factor MCM4 | MCM4 | 1.35E-03 | 4.8 |
| SUMO-activating enzyme subunit 1 | SAE1 | 1.36E-03 | 1.3 |
| Antigen peptide transporter 1 | TAP1 | 1.36E-03 | 2.0 |
| DnaJ homolog subfamily C member 9 | DNAJC9 | 1.37E-03 | 2.6 |
| Centromere protein C 1 | CENPC1 | 1.37E-03 | 1.8 |
| Septin-11 | SEPT11 | 1.39E-03 | 2.8 |
| Replication protein A 70 kDa DNA-binding subunit | RPA1 | 1.40E-03 | 2.2 |
| Ribonucleoside-diphosphate reductase subunit M2 | RRM2 | 1.42E-03 | 2.8 |
| Kanadaptin | SLC4A1AP | 1.42E-03 | 2.8 |
| Treacle protein | TCOF1 | 1.43E-03 | 2.1 |
| Double-stranded RNA-specific adenosine deaminase | ADAR | 1.60E-03 | 3.0 |
| Gap junction alpha-1 protein | GJA1 | 1.67E-03 | -3.0 |
| Nucleoprotein TPR | TPR | 1.73E-03 | 1.5 |
| Serpin H1 | SERPINH1 | 1.81E-03 | 3.4 |
| GMP synthase [glutamine-hydrolyzing] | GMPS | 1.83E-03 | 3.5 |
| Pre-mRNA-splicing factor ISY1 homolog | ISY1 | 1.88E-03 | 2.4 |
| Pre-mRNA 3'-end-processing factor FIP1 | FIP1L1 | 1.88E-03 | 2.4 |
| Drebrin | DBN1 | 2.27E-03 | 3.5 |
| Cluster of V-type proton ATPase subunit B, brain isoform | ATP6V1B2 | 2.33E-03 | 0.9 |
| Procollagen-lysine,2-oxoglutarate 5-dioxygenase 1 | PLOD1 | 2.33E-03 | 3.6 |
| Regulator of chromosome condensation | RCC1 | 2.38E-03 | 2.7 |
| Annexin A3 | ANXA3 | 2.54E-03 | 1.8 |
| Pre-mRNA-processing factor 6 | PRPF6 | 2.56E-03 | 3.0 |
| Apoptosis-inducing factor 1, mitochondrial | AIFM1 | 2.65E-03 | 1.2 |
| Cluster of Alpha-actinin-1 | ACTN1 | 2.72E-03 | 0.7 |
| WD repeat and HMG-box DNA-binding protein 1 | WDHD1 | 2.74E-03 | 2.1 |
| HIV Tat-specific factor 1 | HTATSF1 | 2.75E-03 | 2.1 |
| NADH-cytochrome b5 reductase 1 | CYB5R1 | 2.79E-03 | 2.0 |
| Protein MANBAL | MANBAL | 2.91E-03 | -1.0 |
| Sorting nexin-2 | SNX2 | 2.98E-03 | 2.2 |
| Unconventional myosin-Ie | MYO1E | 3.00E-03 | 2.2 |
| Calreticulin | CALR | 3.25E-03 | 1.1 |
| Poly [ADP-ribose] polymerase 1 | PARP1 | 3.29E-03 | 1.6 |
| Fascin | FSCN1 | 3.33E-03 | 1.4 |
| Fragile X mental retardation protein 1 | FMR1 | 3.63E-03 | 2.5 |
| Sodium/potassium-transporting ATPase subunit beta-1 | ATP1B1 | 3.94E-03 | 3.1 |
| U2 snRNP-associated SURP motif-containing protein | U2SURP | 4.15E-03 | 3.4 |
| Filamin-A | FLNA | 4.19E-03 | 0.9 |
| Solute carrier family 2, facilitated glucose transporter member 1 | SLC2A1 | 4.20E-03 | 2.3 |
| A-kinase anchor protein 13 | AKAP13 | 4.25E-03 | 3.2 |
| RNA-binding protein 39 | RBM39 | 4.44E-03 | 2.0 |
| Replication factor C subunit 2 | RFC2 | 4.58E-03 | 2.6 |
| Brain-specific angiogenesis inhibitor 1-associated protein 2-like protein 1 | BAIAP2L1 | 4.58E-03 | 2.6 |
| Negative elongation factor E | NELFE | 4.58E-03 | 2.6 |
| 78 kDa glucose-regulated protein | HSPA5 | 4.64E-03 | 0.7 |
| Intercellular adhesion molecule 1 | ICAM1 | 4.75E-03 | 3.6 |
| Transferrin receptor protein 1 | TFRC | 4.91E-03 | 3.3 |
| Integrin alpha-2 | ITGA2 | 5.21E-03 | 2.7 |
| Structural maintenance of chromosomes protein 2 | SMC2 | 5.36E-03 | 2.9 |
| Phospholipase A-2-activating protein | PLAA | 5.40E-03 | 2.8 |
| Replication protein A 32 kDa subunit | RPA2 | 5.42E-03 | 3.0 |
| Cluster of Keratin, type II cytoskeletal 5 | KRT5 | 5.54E-03 | -0.5 |
| Nicalin | NCLN | 5.59E-03 | 2.8 |
| Zinc finger RNA-binding protein | ZFR | 5.65E-03 | 3.7 |
| E3 ubiquitin-protein ligase RNF213 | RNF213 | 5.93E-03 | 2.5 |
| Keratin, type I cytoskeletal 23 | KRT23 | 5.93E-03 | 2.7 |
| Extended synaptotagmin-2 | ESYT2 | 6.08E-03 | 2.3 |
| Phospholipid scramblase 1 | PLSCR1 | 6.08E-03 | 2.3 |
| Cluster of Receptor-type tyrosine-protein phosphatase F | PTPRF | 6.49E-03 | 3.0 |
| DNA replication licensing factor MCM5 | MCM5 | 7.08E-03 | 2.9 |
| Cluster of Cyclin-dependent kinase 13 | CDK13 | 3.41E-02 | 1.1 |
